# Epigenetic control of mobile DNA as an interface between experience and genome change

**DOI:** 10.3389/fgene.2014.00087

**Published:** 2014-04-25

**Authors:** James A. Shapiro

**Affiliations:** Department of Biochemistry and Molecular Biology, University of ChicagoChicago, IL, USA

**Keywords:** mutation, evolution, natural genetic engineering, mobile DNA, viruses, mobile genetic elements, non-coding RNA

## Abstract

Mobile DNA in the genome is subject to RNA-targeted epigenetic control. This control regulates the activity of transposons, retrotransposons and genomic proviruses. Many different life history experiences alter the activities of mobile DNA and the expression of genetic loci regulated by nearby insertions. The same experiences induce alterations in epigenetic formatting and lead to trans-generational modifications of genome expression and stability. These observations lead to the hypothesis that epigenetic formatting directed by non-coding RNA provides a molecular interface between life history events and genome alteration.

## Introduction

Understanding the functional organization of the genome and its evolutionary history are key goals of modern molecular biology. The task has become more interesting and complex as we learn more the details of cell processes involving the genome. Recent applications of high resolution technologies to genome expression in animals reveal a dynamic four-dimensional interactive control architecture incompatible with prior notions that genomes contain discrete functional segments of DNA (“genes”) (Mercer and Mattick, 2013). This review will focus on the role of epigenetic regulation of viruses and mobile genetic elements as a key interface between the activities of these agents of evolutionary change and inputs from cell and organism life histories. The hypothesis developed as a result of the review is that disruption of epigenetic silencing constitutes a major target for life history activation of cellular functions for genome change. This likely occurs after genome replication, possibly by changes in small non-coding (snc) RNAs, typically on the order of 20–50 nucleotides long.

## Mobile DNA is a major and functionally significant component of genomes

One of the major surprises to come from the initial sequencing of the human genome was the high abundance of dispersed mobile repeat elements (Consortium, 2001). Today, we estimate that at least two-thirds of our genomes is composed of mobile DNA (De Koning et al., 2011). The human genome is not exceptional in its high content of mobile DNA (http://shapiro.bsd.uchicago.edu/TableII.1.shtml).

We increasingly recognize that viruses contribute to cell genomes (Kokosar and Kordis, 2013). They provide sequences for non-coding ncRNAs (Frias-Lasserre, 2012), sites for transcriptional control (Peaston et al., 2004; Dunn et al., 2005; Maksakova et al., 2006; Conley et al., 2008), and elements important in epigenetic regulation (Brunmeir et al., 2010; Conley and Jordan, 2012). Similar transcriptional and epigenetic regulatory contributions are made by mobile genetic elements (http://shapiro.bsd.uchicago.edu/Table5C-1.MobileElementsFoundtobeExaptedascis-RegulatoryControlSitesinAnimals.html) (Youngson et al., 2005; Kinoshita et al., 2007; Suzuki et al., 2007; Fujimoto et al., 2008; Gehring et al., 2009; Pask et al., 2009; Nakayashiki, 2011).

Mobile DNA is a major source of novel coding information. One mechanism is the process known as “exonization,” when splice signals are utilized in newly inserted DNA segments (http://shapiro.bsd.uchicago.edu/Origin_of_New_Protein_Domains.html). New coding sequences also form by reverse transcription of processed RNAs and genome insertion of the cDNAs, sometimes producing chimeric fusions with existing exons (http://shapiro.bsd.uchicago.edu/Table 5B. Reports of retrogenes in plant and animal genomes.html) (Long, 2001; Betrán et al., 2002; Fu et al., 2010).

It is now clear that mobile genetic elements play a key role in establishing and rewiring genomic networks (http://shapiro.bsd.uchicago.edu/Table5C-1.MobileElementsFoundtobeExaptedascis-RegulatoryControlSitesinAnimals.html) (Feschotte, 2008; Lindblad-Toh et al., 2011; Lowe et al., 2011; Testori et al., 2012; Kokosar and Kordis, 2013). Moreover, mobile element proliferation is a key factor in the formation of very large genomes (http://shapiro.bsd.uchicago.edu/Genome_Size.html).

The potential functional importance of distributed mobile DNA in genomes grows rapidly as evidence accumulates for pervasive genome transcription (http://shapiro.bsd.uchicago.edu/PervasiveGenomeTranscription.html) and for the regulatory role of non-coding RNAs (ncRNAs) in genome expression, including the functional juxtaposition of distant genome regions to activate transcription (http://shapiro.bsd.uchicago.edu/NonCodingRNAinGenomeExpression.html). Mobile elements participate in this long-range genomic communication and provide the sequences of many ncRNAs (Kapusta et al., 2013).

## Cells use RNA-targeted epigenetic control to inhibit the activity of mobile DNA

Given the high content of mobile DNA in many genomes, an important question is: what prevents all the mobility systems from destroying genome integrity? In eukaryotic cells, a major control mechanism is sncRNA-directed epigenetic formatting into silent chromatin (Law and Jacobsen, 2010; Castel and Martienssen, 2013).

Both prokaryotes and eukaryotes have systems for capturing fragments from invading DNA molecules and placing the fragments into special loci encoding sncRNAs (Dumesic and Madhani, 2014). In prokaryotes, these loci are called CRISPRs (clustered regular interspersed palindromic repeats) (http://shapiro.bsd.uchicago.edu/CRISPRs.html) (Marraffini and Sontheimer, 2010; Garrett et al., 2011; Bikard and Marraffini, 2013; Watanabe et al., 2013). The RNA transcripts from CRISPRs are processed into sncRNAs that target cleavage of homologous invading DNA and also inactivation of complementary mRNA (Djordjevic et al., 2012). The details of the RNA processing and interference activities are well-characterized, but the acquisition of DNA fragments is poorly understood. The process must be very rapid, because viral infection yields cells that survive the initial infection with appropriate fragments added to their CRISPR repertoire (Barrangou et al., 2007).

Virtually all eukaryotes investigated, with the notable exception of budding yeast, have mechanisms for sncRNA-directed chromatin silencing. They are based on members of the Argonaute family of sncRNA-processing proteins (http://shapiro.bsd.uchicago.edu/microRNA-directedchromatinsilencing.html). Plants and animals have independently evolved distinct mechanisms of processing the sncRNAs for the Argonaute family systems, but both groups use targeted epigenetic regulatory processes to defend against virus infection (Ding and Voinnet, 2007; Csorba et al., 2009) and prevent genome instability (Table 1). Like prokaryotes, *Drosophila* has specific genomic loci where it acquires fragments of invading DNA to encode the targeting sncRNAs (Brennecke et al., 2007, 2008; Handler et al., 2013).

**Table 1:** **Genome immunity by sncRNA targeting of mobile DNA (see also http://shapiro.bsd.uchicago.edu/TableII.9.shtml for earlier references)**.

**Organisms**	**sncRNA targets**	**References**
Plants	Transposable elements	Rigal and Mathieu, 2011; Ng et al., 2012; Nuthikattu et al., 2013
*Arabidopsis*	Retrotransposons	Mirouze et al., 2009; Slotkin, 2010
Rice	Retrotransposons	Tian et al., 2011
*Brassica*	Retrotransposons	Zhang et al., 2013
*Arabidopsis*	Transposable elements	Mccue et al., 2012
Maize	Transposable elements	Barber et al., 2012; He et al., 2013
Plants	Viruses and viroids	Navarro et al., 2009; Pantaleo, 2011; Zhu and Guo, 2012; Ramesh et al., 2014
Rice, tobacco and *Laodelphgax striatellus* insect vector	Rice stripe virus	Xu et al., 2012b
*Arabidopsis*	Geminiviruses	Vanitharani et al., 2005; Raja et al., 2014
*Caenorhabditis elegans* germ-line	Transposons	Sijen and Plasterk, 2003; Buckley et al., 2012; Lee et al., 2012
*Drosophila*	Viruses	Van Rij et al., 2006
*Drosophila* somatic cells	Retrotransposons	Kawamura et al., 2008
*Drosophila* male germ-line	Retrotransposons	Kalmykova et al., 2005
*Drosophila* female germ-line	Transposons, retrotransposons and retroviruses	Brennecke et al., 2007, 2008
*Drosophila* female germ-line	Telomeric retrotransposons	Shpiz et al., 2009
*Drosophila* gonads	Transposons	Sienski et al., 2012
*Drosophila* somatic and germ-line cells	Transposons, retrotransposons and retroviruses	Handler et al., 2013
*Drosophila* tissue culture cells	Transposons	Chung et al., 2008
Shrimp	White spot syndrome DNA virus	Huang and Zhang, 2013; Sabin and Cherry, 2013
Mammalian cells	EMCV and NoV RNA viruses	Maillard et al., 2013
Human tissue culture cells	LINE retrotransposons	Yang and Kazazian, 2006

## Life history events destabilize genomes and activate mobile DNA

Anyone who has studied real-time genome changes quantitatively knows that mutation frequencies depend upon the treatment of the experimental organism prior to measurement. A wide variety of life history events influence the natural genetic engineering (NGE) functions that generate mutations, especially mobile elements (Table 2; Shapiro, 2011). In some cases, the genome instabilities are large scale and last multiple cell or organismal generations.

**Table 2:** **Life history events that lead to genome destabilization (see also http://shapiro.bsd.uchicago.edu/TableII.8.shtml for earlier references)**.

**Organism**	**Life history event**	**Genome instability**	**References**
Plant	Polyploidization	Transposon and retrotransposon activation	Bento et al., 2013
Rice	Introgression from wild rice (*Zizania*)	Genome-wide variation of all kinds, including transposon reactivation and transgenerational mobile element activation	Wang et al., 2009, 2010, 2013b
Apple	Polyploidization	Aneuploidy	Considine et al., 2012
*Brassica*	Intertribal hybridization; genome triplication; allopolyploidization	Retrotransposition; loss of tandem arrays; Homoeologous shuffling and chromosome compensation	Xiong et al., 2011; Fang et al., 2012; Zhang et al., 2013
Wheat, rye	Allopolyploidization	Loss of repetitive and non-coding DNA, including chromosome-specific sequences; rearrangement of syntenic blocks; transposon and retrotransposon activity	Bento et al., 2008, 2010, 2013; Kraitshtein et al., 2010; Yaakov and Kashkush, 2011b, 2012; Feldman and Levy, 2012; Tomas et al., 2012; Luo et al., 2012; Martis et al., 2013
Sunflower	Polyploidization	Chromosome rearrangements	Lim et al., 2008; Chester et al., 2012
Plants	Polyploidization	Rapid genome reshuffling	Tayale and Parisod, 2013
Plants	Polyploidization	Meiotic and fertilization abnormalities	Grandont et al., 2013
Animals	Polyploidization	Meiotic and fertilization abnormalities	Bogart and Bi, 2013; Choleva and Janko, 2013; Stenberg and Saura, 2013
*Squalius alburnoides* (Cyprinid fish)	Polyploidization	Rapid genome reshuffling; mobile element activity	Collares-Pereira et al., 2013
*Arabidopsis*	Oilseed rape mosaic virus infection	Increased homologous recombination	Yao et al., 2013
*Arabidopsis*	Heat shock	Transgenerational ONSEN retrotransposon activation	Matsunaga et al., 2012
*Arabidopsis*	Volatiles from UV-irradiated *Arabidopsis* or tobacco plants	Increased homologous recombination	Yao et al., 2011
*Arabidopsis*	Abiotic stresses (ionizing radiation, heavy metals, chlorine, temperature and water)	Somatic and heritable changes in homologous recombination, strand breakage	Boyko et al., 2010; Rahavi et al., 2011; Yao and Kovalchuk, 2011
Tobacco	Tobacco mosaic virus infection	Increased homologous recombination	Kathiria et al., 2010
Rice	Tissue culture cultivation	Genomic DNA fragment length polymorphisms	Wang et al., 2013a
Rice	Etoposide exposure	Increased transposon activity	Yang et al., 2012
Human	Human papillomavirus (HPV) integration	Extensive rearrangements, often focused on insertion site	Korzeniewski et al., 2011; Akagi et al., 2014

Many observations demonstrate responses of the circuits controlling NGE functions to biological and abiotic inputs. It is particularly significant that many such responses occur following exceptional cell interactions with viruses or with other cells, either by infection or by hybridization (Table 2). As we might expect, the introduction of alien DNA or chromatin into a cell often has disruptive effects on genome homeostasis (Shapiro, 2014).

## Epigenetic changes in response to life history events

One of the most active research areas in the second decade of the 21st century is analyzing the impact of life history events on the epigenetic layers of cell regulatory architecture (Table 3) (Chinnusamy and Zhu, 2009a; Vandegehuchte and Janssen, 2013). The observed epigenetic responses include alterations to cytosine methylation in DNA (Chinnusamy and Zhu, 2009b), histone modifications in nucleosomes, and sncRNAs (Ruiz-Ferrer and Voinnet, 2009; Ng et al., 2012) as well as transgenerational inheritance of complex novel phenotypes (Zucchi et al., 2012), frequently induced by stress (Boyko and Kovalchuk, 2010). The phenomenon of hybrid vigor, or heterosis, increasingly is viewed as an alteration in sncRNA-targeted epigenetic formatting stimulated by the encounter of two distinct genome control regimes (Groszmann et al., 2011; Miller et al., 2012; Shivaprasad et al., 2012).

**Table 3:** **Life history events that induce epigenetic changes (see also http://shapiro.bsd.uchicago.edu/TableII.10.shtml for earlier references)**.

**Organism**	**Life history event**	**Epigenetic change**	**References**
Plants	Hybridization, polyploidization	sncRNA changes	Ng et al., 2012
Maize	Hybridization	rasRNA variation	Barber et al., 2012
Cotton	Allotetraploidization	Changes in mi- and siRNA content and levels	Pang et al., 2009
*Brassica napus*	Intertribal hybridization and introgression	Changes in cytosine methylation	Zhang et al., 2013
Wheat	Allopolyploidization	Multigenerational transposon methylation changes	Kraitshtein et al., 2010; Yaakov and Kashkush, 2011a,b
Wheat	Hybridization and polyploidization	Deregulation of sncRNAs	Kenan-Eichler et al., 2011
*Solanaceae*	Interspecific grafting	DNA methylation changes	Wu et al., 2013
Tobacco	Geminivirus and geminivirus-beta satellite infection	Suppression of DNA methylation-base silencing	Vanitharani et al., 2005; Buchmann et al., 2009; Yang et al., 2011
Tobacco	Tobacco mosaic virus infection	Heritable resistance to viral, bacterial and fungal pathogens	Kathiria et al., 2010
Rice	Drought exposure	Multigenerational DNA methylation changes	Zheng et al., 2013
Rice	Nitrogen deprivation	Heritable stress tolerance	Kou et al., 2011
Rice	Tissue culture cultivation	DNA methylation changes	Fukai et al., 2010; Wang et al., 2013a
Rice	Etoposide exposure	Multigenerational DNA methylation changes	Yang et al., 2012
Rice	Salt exposure	DNA methylation changes	Karan et al., 2012
Rice	Heavy metal exposure	Multigenerational DNA methylation changes	Ou et al., 2012
Rice	Abiotic stresses	Novel sncRNAs in the infloresences	Barrera-Figueroa et al., 2012
Pear seeds	Desiccation	DNA methylation changes	Michalak et al., 2013
*Arabidopsis*	Interspecific hybridization	Polycomb response complex changes	Burkart-Waco et al., 2013
*Arabidopsis*	Geminivirus (Cabbage leaf curl virus, CaLCuV) infection	Epigenetic silencing	Aregger et al., 2012
*Arabidopsis*	Stress response	Alteration of *Athila* family retrotransposon sncRNA	Mccue et al., 2012
*Arabidopsis*	Biotic stresses (bacteria, hormones)	Increased DNA methylation	Dowen et al., 2012
*Arabidopsis*	β-amino-butyric acid	Imprinted resistance (multigenerational) to *Pseduomonas syringae* and *Hyaloperonospora arabidopsidis* fungus	Slaughter et al., 2012
*Arabidopsis*	Salt exposure	DNA methylation, nucleosome composition	Bilichak et al., 2012
*Arabidopsis*	Hyperosmotic priming	Shortening and fractionation of H3K27me3 islands	Sani et al., 2013
Wild rye	Abiotic stresses	DNA methylation	Yu et al., 2013b
Neptune grass	Cadmium	DNA methylation and chromatin patterning	Greco et al., 2012
Plant and mammalian cells	Cadmium	DNA methylation and histone modification	Wang et al., 2012
Nematode (*Caenorhabditis elegans*)	Flock house virus expression	Transgenerational resistance transmitted by sncRNAs	Rechavi et al., 2011
Mosquito (*Aedes aegypti*)	*Wolbachia* infection	Disruption of cytosine methylation	Ye et al., 2013
Carp	Allotetraploidization	Localized hypermethylation	Xiao et al., 2013
*Squalius alburnoides* (fish)	Polyploidization	Alterations in sncRNA patterns	Inacio et al., 2012
Rats	Exposure to dioxin and endocrine disruptors of F0 generation	Transgenerational inheritance of adult onset diseases and sperm epimutations	Manikkam et al., 2012, 2013
Rats	Vinclozolin fungicide exposure of F0 males	Transgeneration changes to physiology, behavior, metabolic activity, and transcriptome in discrete brain nuclei, altered restraint stress responses	Crews et al., 2012
Pigs	Diet supplementation of F0 with methylating micronutrients	Transgenerational inheritance of extra fat and DNA methylation changes	Braunschweig et al., 2012
Mouse neuronal cells	Short-term hypoxia	DNA methylation changes	Hartley et al., 2013
Humans	High fat diet	DNA methylation changes	Jacobsen et al., 2012
Humans	Early life trauma	DNA methylation changes	Labonte et al., 2012
Humans	Cadmium	DNA hypo-methylation	Hossain et al., 2012
Human lymphocytes	Epstein-Bar virus (EBV) infection	Hypermethylation of tumor suppressor loci, DNA methylation changes	Leonard et al., 2011; Kaneda et al., 2012; Queen et al., 2013
Human liver cells	Hepatitis B virus infection	DNA methylation, histone and sncRNA changes	Tian et al., 2013; Rongrui et al., 2014
Gastric epithelium	*Helicobacter pylori* infection	DNA methylation and histone changes	Ding et al., 2010; Alvarez et al., 2013; Chiariotti et al., 2013
Schwann cells	*Mycobacterium leprae* infection	Reprogramming to stem cell-like state	Masaki et al., 2013

Many of the studies demonstrating induced epigenetic modifications also document accompanying genome instabilities and emphasize their evolutionary potential (Madlung and Wendel, 2013). It is noteworthy that many of the same stimuli are involved in both genomic and epigenomic responses in plants (Hegarty et al., 2013) and animals (Arkhipova and Rodriguez, 2013). The common stimuli include infection and symbiosis (Hamon and Cossart, 2008; Bierne et al., 2012; Takahashi, 2014), hybridization and changes in ploidy.

## Direct interactions between NGE activities and epigenetic regulatory functions

In addition to disruption of sncRNA-targeted inhibition, there is limited but growing evidence that NGE functions acting on DNA molecules interact directly with epigenetic control factors. There is convincing evidence of the connection between NGE and the epigenome in DNA damage repair and retroviral or retrotransposon insertions into chromosomes.

### Epigenetic involvement in DNA proofreading and repair

There are recent reports that a specific histone modification (H3K36me3) primes DNA mismatch repair (Schmidt and Jackson, 2013), that H3K56 acetylation affects mismatch repair (Kadyrova et al., 2013), that hypoacetylation of H3K56 by HDACs 1 and 2 facilitates recruitment of non-homologous end-joining (NHEJ) proteins (Miller et al., 2010; Munoz-Galvan et al., 2013), and that nucleosome remodeling is integral to DS break repair (Seeber et al., 2013). Longstanding observations document the involvement of a specific histone, gamma-H2AX, in DS break repair and NHEJ (Kinner et al., 2008; Altaf et al., 2009; Dickey et al., 2009b; Redon et al., 2009; Firsanov et al., 2011; Chen et al., 2013). A direct role in chromatin remodeling for DNA repair has been claimed for another H2 analog, H2A.Z (Xu et al., 2012a).

Published reports indicate that H2AX incorporation into chromatin suppresses conversion of single-strand nicks to DS breaks (Franco et al., 2006) and affects the processing of the ends of broken DNA molecules (Helmink et al., 2011). H2AX operates in phosphorylated form (Rogakou et al., 1998; Kinner et al., 2008).

Beyond the role of H2AX, chromatin dynamics play an essential role in DNA repair and genome homeostasis (Lahue and Frizzell, 2012; Shi and Oberdoerffer, 2012). Many reports claim repair roles for chromatin regulators, remodeling complexes and nucleosome exchange factors (Ryan and Owen-Hughes, 2011):

– in DNA damage tolerance (Conaway and Conaway, 2009; Falbo et al., 2009);
– after exposure to ionizing radiation (Hunt et al., 2013);
– in UV damage responses (Palomera-Sanchez and Zurita, 2011; Yu et al., 2011);
– in DS break repair by NHEJ and HR (Van Attikum and Gasser, 2005; Williams et al., 2007; Robert et al., 2011; Xu and Price, 2011; Price and D'andrea, 2013);
– in PolyADP-dependent DNA repair (Ahel et al., 2009);
– in NER as well as DS break repair (Osley et al., 2007; Czaja et al., 2012; Yu et al., 2013a).

Nucleosome disassembly is probably necessary for certain repair processes (Linger and Tyler, 2007; Amouroux et al., 2010; Gospodinov and Herceg, 2013), and histone modifications affect damage-induced checkpoint signaling (Chen and Tyler, 2008). Once repair is complete, nucleosome modifications are reversed, and H2AX~P is eliminated from chromatin (Svetlova et al., 2010). So-called “bystander” cells, which are not subjected to DNA damage but are in the same culture as irradiated cells, also display H2AX phosphorylation (Sokolov et al., 2007; Dickey et al., 2009a, 2011).

A key feature of genome repair is that H2AX-marked damaged DNA mobilizes to subnuclear “repair centers” where homologous recombination and NHEJ proteins also localize (Lisby and Rothstein, 2005; Plate et al., 2008; Bekker-Jensen and Mailand, 2010). A role for chromatin in mobilization of damaged DNA has been proposed (Seeber et al., 2013), but multiple sources of evidence are lacking.

### Retroviral and retrotransposon integrases

A more extensive case for NGE-chromatin interactions comes from analysis of retroviral and retrotransposon insertion specificities (Zhang and Mager, 2012). Each type of retrovirus displays a characteristic insertion specificity for its provirus (Lewinski et al., 2006). A number of targeting mechanisms involve epigenetic formatting molecules.

In budding yeast, Ty1 retrotransposon integrase contacts an H2A/H2B interface upstream of RNA polymerase III initiation sites (Baller et al., 2012; Bridier-Nahmias and Lesage, 2012; Mularoni et al., 2012). Histone deacetylase Hos2 and Trithorax group protein Set3 stimulate this nucleosome-targeted integration (Mou et al., 2006), and chromatin remodeling factor Isw2p is also implicated (Bachman et al., 2005). In contrast, the Ty5 retrotransposon inserts in silent chromatin, targeted by binding of its integrase to the Sir4 heterochromatin nucleating factor (Xie et al., 2001; Dai et al., 2007; Brady et al., 2008; Baller et al., 2011).

HIV and other lentiviral targeted integration into actively transcribed regions of the genome is associated with transcription-associated histone modifications, including H3 acetylation, H4 acetylation, and H3 K4 methylation, but is disfavored in regions rich in transcription-inhibiting modifications, which include H3K27me3 and DNA CpG methylation (Wang et al., 2007). The specificity results from integrase tethering by the LEDGF/p75 chromatin-binding growth factor (Vanegas et al., 2005; Llano et al., 2006; Ciuffi, 2008; Meehan and Poeschla, 2010; Zheng et al., 2010; Christ and Debyser, 2013). Replacing the LEDGF/p75 domain that interacts with expressed chromatin by the CBX1 domain, which binds histones H3K9me2 or H3K9me3 found in pericentric heterochromatin, targets HIV insertions to silent chromatin regions (Gijsbers et al., 2010).

Murine leukemia virus (MuLV) insertion targeting to initiation sites upstream of actively transcribed regions involves integrase interactions with bromodomain proteins BRD2, BRD3, and BRD4 (De Rijck et al., 2013; Gupta et al., 2013; Sharma et al., 2013a). Interestingly, chromatin recognition bromodomain protein BRD4 antagonizes HIV provirus reactivation (Zhu et al., 2012).

Certain retrotransposons are specifically targeted to centromeres (Wolfgruber et al., 2009; Birchler and Presting, 2012; Tsukahara et al., 2012; Sharma et al., 2013b), which have a special chromatin configuration characterized by centromeric versions of H3 (Henikoff and Dalal, 2005; Vos et al., 2006; Partridge, 2008; Zhang et al., 2008a). Centromeric retrotransposons in rice are highly associated with H3K9me2, a hallmark for heterochromatin (Neumann et al., 2007). Some centromeric retrotransposons encode integrase proteins with histone-binding chromodomains at their carboxy-termini (Neumann et al., 2011). Chromodomains recognize lysine methylation (Blus et al., 2011; Yap and Zhou, 2011; Eissenberg, 2012).

It is probably not coincidental that the most widely distributed group of retrotransposons among all eukaryotic clades are the “chromoviruses,” which are so named because they have chromodomains in their integrase proteins (Gorinsek et al., 2004; Kordis, 2005; Novikov et al., 2012; Weber et al., 2013). A chromodomain has been reported to target fungal chromovirus MAGGY insertions to heterochromatin marked by H3K9me2/me3 (Gao et al., 2008). An integrase chromodomain also participates in activator protein-targeted insertion of fission yeast retrotransposon Tf1 upstream of RNA polymerase II transcription start sites (Hizi and Levin, 2005; Chatterjee et al., 2009).

### DNA transposons

In contrast with many retrotransposons that interact with nucleosomes, the DNA transposon Hermes inserts preferentially in budding yeast into nucleosome-free regions of the genome (Gangadharan et al., 2010). The widely used P element DNA transposons in *Drosophila* show targeting (called “P element homing”) by incorporating binding sites for various regulatory factors, including chromatin insulators (Bender and Hudson, 2000; Fujioka et al., 2009) and Polycomb group response elements (Kassis, 2002; Cheng et al., 2012).

## Epigenetic reformatting after DNA replication and ncRNAs as potential agents for transmitting experience to the genome

While the evidence is increasingly abundant for effects of different life history events on epigenetic regulation in general, and on genome homeostasis in particular, it is far from clear how those effects occur (Lim and Brunet, 2013). We know very little about the connections between cell sensors and epigenetic (re)formatting complexes (Erdel et al., 2011; Narlikar et al., 2013). Deciphering those connections is currently an important research goal.

DNA replication provides a key decision point for maintaining or changing chromatin configurations (Poot et al., 2005; Liu and Gong, 2011; Mermoud et al., 2011). The replication apparatus must disassemble chromatin for polymerization and then reassemble chromatin once replication is complete. Replication takes place only in dividing cells, and transgenerational inheritance of epigenetic states must involve the proliferating cells that give rise to gametes. Transfer of outside information from somatic tissues to the germline has been reported in mammals (Sharma, 2013; Skinner et al., 2013). And epigenetic windows of susceptibility to environmental insults have been suggested during sperm development (Soubry et al., 2014). Since there is no segregated germ line in plants and eukaryotic microbes, the same cells that experience environmental inputs can also be the progenitors of gametes.

A number of different factors have been found or hypothesized to participate in post-replication chromatin restoration: histone chaperones (Budhavarapu et al., 2013), RNA editing and sncRNAs (Savva et al., 2013), chromatin remodeler SMARCAD1 (Mermoud et al., 2011), chromatin assembly factor 1 (Huang and Jiao, 2012), histone chaperon FACT (Winkler and Luger, 2011) and Swi/Snf complexes (Neves-Costa and Varga-Weisz, 2006; Ryan and Owen-Hughes, 2011; Zhu et al., 2013), and ISW1 complexes (Erdel and Rippe, 2011).

One frequently overlooked feature of post-replication reestablishment of epigenetic formatting is where in the nucleus it might occur. Replication takes place in specialized “replication factories” (Vago et al., 2009; Guillou et al., 2010). Does chromatin reestablishment occur in the same location or does it involve migration of newly replicated DNA segments to distinct subnuclear “chromatin factories,” like the ones that exist in the nucleolus for heterochromatin formation on rRNA-encoding DNA (Guetg and Santoro, 2012)? If so, such post-replication relocalization would be guided by the nucleoskeleton and lncRNAs (Mercer and Mattick, 2013; Mercer et al., 2013) and might present an attractive target for stress response and sensory input signaling (Weiner et al., 2012).

It is notable that changes to ncRNAs are frequently cited with regard to the impact of life history events on the genome (Sunkar et al., 2007; Khraiwesh et al., 2012; Lelandais-Briere et al., 2012; Nakaminami et al., 2012; Amaral et al., 2013). In the plant literature, there is documentation of numerous ncRNA changes in response to particular biotic and abiotic stress regimes (Table 4).

**Table 4:** **Changes in non-coding RNAs in response to life history events**.

**Stress or input**	**Organism(s)**	**References**
Salt	Multiple plants	Ding et al., 2009; Qin et al., 2011; Macovei and Tuteja, 2012; Carnavale Bottino et al., 2013; Li et al., 2013; Ren et al., 2013; Zhuang et al., 2014
Drought	Multiple plants	Barrera-Figueroa et al., 2011; Li et al., 2011a; Qin et al., 2011; Wang et al., 2011; Eldem et al., 2012; Ferreira et al., 2012; Ding et al., 2013; Gentile et al., 2013; Shuai et al., 2013
Waterlogging	Maize, poplar	Zhang et al., 2008b; Liu et al., 2012; Ren et al., 2012; Zhai et al., 2013
Cold stress	Wheat	Tang et al., 2012
Aluminum	Soybeans	Chen et al., 2012a; Zeng et al., 2012
Cadmium	Radish	Xu et al., 2013
Boron	Barley	Ozhuner et al., 2013
ethylene	*Medicago truncatula* (barrel clover)	Chen et al., 2012b
Ozone	*Arabidopsis*	Iyer et al., 2012
Hypoxia	*Arabidopsis*	Moldovan et al., 2010
Low phosphorous	Maize	Zhang et al., 2012
Low nitrate	Maize	Xu et al., 2011
Sulfur deprivation	*Chlamydomonas reinhardtii*	Shu and Hu, 2012
Abiotic stresses	Multiple plants	Kulcheski et al., 2011; Li et al., 2011b; Barrera-Figueroa et al., 2012; Sun et al., 2012; Zhan et al., 2012; Ballen-Taborda et al., 2013
Physiological stressors and invasive plant infection	Rice blast fungus, *Magnaporthe oryzae*	Raman et al., 2013
Virus infection	Multiple plants, rice	Du et al., 2011; Sha et al., 2014
Viral and bacterial infections	Multiple plants, cassava (*Xanthomonas* infection)	Perez-Quintero et al., 2012; Zvereva and Pooggin, 2012; Pelaez and Sanchez, 2013; Quintero et al., 2013
Bacterial/phytoplasma infection	Multiple plants, lime trees	Zhang et al., 2011; Ehya et al., 2013
Powdery mildew infection	Wheat	(Xin et al., 2011) miRNAs (Xin et al., 2010)
*Verticillium* wilt infection	Cotton, eggplant	Yin et al., 2012; Yang et al., 2013

A number of observations about resistance to biotic and abiotic stresses are consistent with a key role for ncRNA changes in life history responses. Several viruses encode siRNA suppressors to overcome host defenses (Jiang et al., 2012; Omarov and Scholthof, 2012; Guo and Lu, 2013). Transgenic constructs encoding constitutive miRNA expression can lead to salt and drought tolerance in creeping bentgrass (Zhou et al., 2013), to immunity against blast fungus in rice (Li et al., 2014), and in *Arabidopsis* to greater salt and alkalinity sensitivity (Gao et al., 2011). Acquired aphid resistance in *Arabidopsis* involves sncRNA changes (Kettles et al., 2013), and most acquired stress resistances in plants display transgenerational epigenetic inheritance (Holeski et al., 2012; Luna and Ton, 2012; Slaughter et al., 2012).

## Speculative conclusions about an epigenetic interface between experience and genome change

Mobile DNA and other NGE functions are the key agents for adaptively significant changes in genome organization and DNA sequences. The data reviewed and tabulated above establish the importance of RNA-directed chromatin formatting in the regulation and operation of mobile elements, viruses and DNA repair functions. In addition, there is a remarkable correlation between the life history events that activate NGE functions to destabilize genomes and those that lead to alteration of chromatin states and DNA methylation patterns.

The preceding observations lead to the plausible hypothesis that epigenetic regulation serves as a key interface between organismal life history and the agents that restructure genomic DNA. This hypothesis is supported by the limited number of cases where empirical observations have established direct molecular connections between NGE functions and components of the epigenetic control system: histones, nucleosomes, and chromatin reformatting complexes.

If, as I expect, further research bolsters the epigenome-NGE correlations and connections documented above, then we need to ask: what components(s) of the epigenetic control apparatus communicate information about experience to NGE operators? We do not know the answer to this fundamental question. However, the data reported in Table 4 indicate that ncRNAs are good candidates for key intermediates in the experience-genome signal transduction process. If this is so, then ncRNAs are logical molecular targets for modulating genome change toward potentially adaptive outcomes. Let us hope that research aimed at examining this proposal deepens our understanding of how life history impacts both epigenetic and genome change operations (Tables 2–4), whether or not my speculation ultimately proves to be correct.

### Conflict of interest statement

The author declares that the research was conducted in the absence of any commercial or financial relationships that could be construed as a potential conflict of interest.

## References

[B1] Ahel D. Horejsi Z. Wiechens N. Polo S. E. Garcia-Wilson E. Ahel I. (2009). Poly(ADP-ribose)-dependent regulation of DNA repair by the chromatin remodeling enzyme ALC1. Science 325, 1240–1243 10.1126/science.1177321 19661379 PMC3443743

[B2] Akagi K. Li J. Broutian T. R. Padilla-Nash H. Xiao W. Jiang B. (2014). Genome-wide analysis of HPV integration in human cancers reveals recurrent, focal genomic instability. Genome Res. 24, 185–199 10.1101/gr.164806.113 24201445 PMC3912410

[B3] Altaf M. Auger A. Covic M. Cote J. (2009). Connection between histone H2A variants and chromatin remodeling complexes. Biochem. Cell Biol. 87, 35–50 10.1139/O08-140 19234522

[B4] Alvarez M. C. Santos J. C. Maniezzo N. Ladeira M. S. Da Silva A. L. Scaletsky I. C. (2013). MGMT and MLH1 methylation in Helicobacter pylori-infected children and adults. World J. Gastroenterol. 19, 3043–3051 10.3748/wjg.v19.i20.3043 23716983 PMC3662943

[B5] Amaral P. P. Dinger M. E. Mattick J. S. (2013). Non-coding RNAs in homeostasis, disease and stress responses: an evolutionary perspective. Brief. Funct. Genomics 12, 254–278 10.1093/bfgp/elt016 23709461

[B6] Amouroux R. Campalans A. Epe B. Radicella J. P. (2010). Oxidative stress triggers the preferential assembly of base excision repair complexes on open chromatin regions. Nucleic Acids Res. 38, 2878–2890 10.1093/nar/gkp1247 20071746 PMC2875005

[B7] Aregger M. Borah B. K. Seguin J. Rajeswaran R. Gubaeva E. G. Zvereva A. S. (2012). Primary and secondary siRNAs in geminivirus-induced gene silencing. PLoS Pathog. 8:e1002941 10.1371/journal.ppat.1002941 23028332 PMC3460622

[B8] Arkhipova I. R. Rodriguez F. (2013). Genetic and epigenetic changes involving (retro)transposons in animal hybrids and polyploids. Cytogenet. Genome Res. 140, 295–311 10.1159/000352069 23899811

[B9] Bachman N. Gelbart M. E. Tsukiyama T. Boeke J. D. (2005). TFIIIB subunit Bdp1p is required for periodic integration of the Ty1 retrotransposon and targeting of Isw2p to S. cerevisiae tDNAs. Genes Dev. 19, 955–964 10.1101/gad.1299105 15833918 PMC1080134

[B10] Ballen-Taborda C. Plata G. Ayling S. Rodriguez-Zapata F. Becerra Lopez-Lavalle L. A. Duitama J. (2013). Identification of cassava MicroRNAs under abiotic stress. Int. J. Genomics 2013, 857986 10.1155/2013/857986 24328029 PMC3845235

[B11] Baller J. A. Gao J. Stamenova R. Curcio M. J. Voytas D. F. (2012). A nucleosomal surface defines an integration hotspot for the Saccharomyces cerevisiae Ty1 retrotransposon. Genome Res. 22, 704–713 10.1101/gr.129585.111 22219511 PMC3317152

[B12] Baller J. A. Gao J. Voytas D. F. (2011). Access to DNA establishes a secondary target site bias for the yeast retrotransposon Ty5. Proc. Natl. Acad. Sci. U.S.A. 108, 20351–20356 10.1073/pnas.1103665108 21788500 PMC3251128

[B13] Barber W. T. Zhang W. Win H. Varala K. K. Dorweiler J. E. Hudson M. E. (2012). Repeat associated small RNAs vary among parents and following hybridization in maize. Proc. Natl. Acad. Sci. U.S.A. 109, 10444–10449 10.1073/pnas.1202073109 22689990 PMC3387101

[B14] Barrangou R. Fremaux C. Deveau H. Richards M. Boyaval P. Moineau S. (2007). CRISPR provides acquired resistance against viruses in prokaryotes. Science 315, 1709–1712 10.1126/science.1138140 17379808

[B15] Barrera-Figueroa B. E. Gao L. Diop N. N. Wu Z. Ehlers J. D. Roberts P. A. (2011). Identification and comparative analysis of drought-associated microRNAs in two cowpea genotypes. BMC Plant Biol. 11:127 10.1186/1471-2229-11-127 21923928 PMC3182138

[B16] Barrera-Figueroa B. E. Gao L. Wu Z. Zhou X. Zhu J. Jin H. (2012). High throughput sequencing reveals novel and abiotic stress-regulated microRNAs in the inflorescences of rice. BMC Plant Biol. 12:132 10.1186/1471-2229-12-132 22862743 PMC3431262

[B17] Bekker-Jensen S. Mailand N. (2010). Assembly and function of DNA double-strand break repair foci in mammalian cells. DNA Repair (Amst.) 9, 1219–1228 10.1016/j.dnarep.2010.09.010 21035408

[B18] Bender W. Hudson A. (2000). P element homing to the Drosophila bithorax complex. Development 127, 3981–3992 Available online at: http://dev.biologists.org/content/127/18/3981.long 10952896 10.1242/dev.127.18.3981

[B19] Bento M. Gustafson P. Viegas W. Silva M. (2010). Genome merger: from sequence rearrangements in triticale to their elimination in wheat-rye addition lines. Theor. Appl. Genet. 121, 489–497 10.1007/s00122-010-1325-6 20383487

[B20] Bento M. Pereira H. S. Rocheta M. Gustafson P. Viegas W. Silva M. (2008). Polyploidization as a retraction force in plant genome evolution: sequence rearrangements in triticale. PLoS ONE 3:e1402 10.1371/journal.pone.0001402 18167561 PMC2151762

[B21] Bento M. Tomas D. Viegas W. Silva M. (2013). Retrotransposons represent the most labile fraction for genomic rearrangements in polyploid plant species. Cytogenet. Genome Res. 140, 286–294 10.1159/000353308 23899810

[B22] Betrán E. Thornton K. Long M. (2002). Retroposed new genes out of the X in Drosophila. Genome Res. 12, 1854–1859 10.1101/gr.6049 12466289 PMC187566

[B23] Bierne H. Hamon M. Cossart P. (2012). Epigenetics and bacterial infections. Cold Spring Harb. Perspect. Med. 2:a010272 10.1101/cshperspect.a010272 23209181 PMC3543073

[B24] Bikard D. Marraffini L. A. (2013). Control of gene expression by CRISPR-Cas systems. F1000Prime Rep. 5:47 10.12703/p5-47 24273648 PMC3816762

[B25] Bilichak A. Ilnystkyy Y. Hollunder J. Kovalchuk I. (2012). The progeny of *Arabidopsis thaliana* plants exposed to salt exhibit changes in DNA methylation, histone modifications and gene expression. PLoS ONE 7:e30515 10.1371/journal.pone.0030515 22291972 PMC3264603

[B26] Birchler J. A. Presting G. G. (2012). Retrotransposon insertion targeting: a mechanism for homogenization of centromere sequences on nonhomologous chromosomes. Genes Dev. 26, 638–640 10.1101/gad.191049.112 22474258 PMC3323874

[B27] Blus B. J. Wiggins K. Khorasanizadeh S. (2011). Epigenetic virtues of chromodomains. Crit. Rev. Biochem. Mol. Biol. 46, 507–526 10.3109/10409238.2011.619164 22023491 PMC3223283

[B28] Bogart J. P. Bi K. (2013). Genetic and genomic interactions of animals with different ploidy levels. Cytogenet. Genome Res. 140, 117–136 10.1159/000351593 23751376

[B29] Boyko A. Golubov A. Bilichak A. Kovalchuk I. (2010). Chlorine ions but not sodium ions alter genome stability of Arabidopsis thaliana. Plant Cell Physiol. 51, 1066–1078 10.1093/pcp/pcq048 20385609

[B30] Boyko A. Kovalchuk I. (2010). Transgenerational response to stress in *Arabidopsis thaliana*. Plant Signal. Behav. 5, 995–998 10.1371/journal.pone.0009514 20724818 PMC3115178

[B31] Brady T. L. Schmidt C. L. Voytas D. F. (2008). Targeting integration of the Saccharomyces Ty5 retrotransposon. Methods Mol. Biol. 435, 153–163 10.1007/978-1-59745-232-8_11 18370074

[B32] Braunschweig M. Jagannathan V. Gutzwiller A. Bee G. (2012). Investigations on transgenerational epigenetic response down the male line in F2 pigs. PLoS ONE 7:e30583 10.1371/journal.pone.0030583 22359544 PMC3281031

[B33] Brennecke J. Aravin A. A. Stark A. Dus M. Kellis M. Sachidanandam R. (2007). Discrete small RNA-generating loci as master regulators of transposon activity in Drosophila. Cell 128, 1089–1103 10.1016/j.cell.2007.01.043 17346786

[B34] Brennecke J. Malone C. D. Aravin A. A. Sachidanandam R. Stark A. Hannon G. J. (2008). An epigenetic role for maternally inherited piRNAs in transposon silencing. Science 322, 1387–1392 10.1126/science.1165171 19039138 PMC2805124

[B35] Bridier-Nahmias A. Lesage P. (2012). Two large-scale analyses of Ty1 LTR-retrotransposon de novo insertion events indicate that Ty1 targets nucleosomal DNA near the H2A/H2B interface. Mob. DNA 3:22 10.1186/1759-8753-3-22 23244340 PMC3545840

[B36] Brunmeir R. Lagger S. Simboeck E. Sawicka A. Egger G. Hagelkruys A. (2010). Epigenetic regulation of a murine retrotransposon by a dual histone modification mark. PLoS Genet. 6:e1000927 10.1371/journal.pgen.1000927 20442873 PMC2861705

[B37] Buchmann R. C. Asad S. Wolf J. N. Mohannath G. Bisaro D. M. (2009). Geminivirus AL2 and L2 proteins suppress transcriptional gene silencing and cause genome-wide reductions in cytosine methylation. J. Virol. 83, 5005–5013 10.1128/jvi.01771-08 19279102 PMC2682068

[B38] Buckley B. A. Burkhart K. B. Gu S. G. Spracklin G. Kershner A. Fritz H. (2012). A nuclear Argonaute promotes multigenerational epigenetic inheritance and germline immortality. Nature 489, 447–451 10.1038/nature11352 22810588 PMC3509936

[B39] Budhavarapu V. N. Chavez M. Tyler J. K. (2013). How is epigenetic information maintained through DNA replication? Epigenetics Chromatin 6:32 10.1186/1756-8935-6-32 24225278 PMC3852060

[B40] Burkart-Waco D. Ngo K. Dilkes B. Josefsson C. Comai L. (2013). Early disruption of maternal-zygotic interaction and activation of defense-like responses in Arabidopsis interspecific crosses. Plant Cell 25, 2037–2055 10.1105/tpc.112.108258 23898028 PMC3723611

[B41] Carnavale Bottino M. Rosario S. Grativol C. Thiebaut F. Rojas C. A. Farrineli L. (2013). High-throughput sequencing of small RNA transcriptome reveals salt stress regulated microRNAs in sugarcane. PLoS ONE 8:e59423 10.1371/journal.pone.0059423 23544066 PMC3609749

[B42] Castel S. E. Martienssen R. A. (2013). RNA interference in the nucleus: roles for small RNAs in transcription, epigenetics and beyond. Nat. Rev. Genet. 14, 100–112 10.1038/nrg3355 23329111 PMC4205957

[B43] Chatterjee A. G. Leem Y. E. Kelly F. D. Levin H. L. (2009). The chromodomain of Tf1 integrase promotes binding to cDNA and mediates target site selection. J. Virol. 83, 2675–2685 10.1128/JVI.01588-08 19109383 PMC2648289

[B44] Chen C. C. Tyler J. (2008). Chromatin reassembly signals the end of DNA repair. Cell Cycle 7, 3792–3797 10.4161/cc.7.24.7188 19066448 PMC5842806

[B45] Chen L. Wang T. Zhao M. Tian Q. Zhang W. H. (2012a). Identification of aluminum-responsive microRNAs in *Medicago truncatula* by genome-wide high-throughput sequencing. Planta 235, 375–386 10.1007/s00425-011-1514-9 21909758

[B46] Chen L. Wang T. Zhao M. Zhang W. (2012b). Ethylene-responsive miRNAs in roots of *Medicago truncatula* identified by high-throughput sequencing at whole genome level. Plant Sci. 184, 14–19 10.1016/j.plantsci.2011.11.007 22284705

[B47] Chen W. T. Alpert A. Leiter C. Gong F. Jackson S. P. Miller K. M. (2013). Systematic identification of functional residues in mammalian histone H2AX. Mol. Cell. Biol. 33, 111–126 10.1128/mcb.01024-12 23109425 PMC3536310

[B48] Cheng Y. Kwon D. Y. Arai A. L. Mucci D. Kassis J. A. (2012). P-element homing is facilitated by engrailed polycomb-group response elements in *Drosophila melanogaster*. PLoS ONE 7:e30437 10.1371/journal.pone.0030437 22276200 PMC3261919

[B49] Chester M. Gallagher J. P. Symonds V. V. Cruz Da Silva A. V. Mavrodiev E. V. Leitch A. R. (2012). Extensive chromosomal variation in a recently formed natural allopolyploid species, *Tragopogon miscellus* (Asteraceae). Proc. Natl. Acad. Sci. U.S.A. 109, 1176–1181 10.1073/pnas.1112041109 22228301 PMC3268322

[B50] Chiariotti L. Angrisano T. Keller S. Florio E. Affinito O. Pallante P. (2013). Epigenetic modifications induced by *Helicobacter pylori* infection through a direct microbe-gastric epithelial cells cross-talk. Med. Microbiol. Immunol. 202, 327–337 10.1007/s00430-013-0301-6 23715627

[B51] Chinnusamy V. Zhu J. K. (2009a). Epigenetic regulation of stress responses in plants. Curr. Opin. Plant Biol. 12, 133–139 10.1016/j.pbi.2008.12.006 19179104 PMC3139470

[B52] Chinnusamy V. Zhu J. K. (2009b). RNA-directed DNA methylation and demethylation in plants. Sci. China C Life Sci. 52, 331–343 10.1007/s11427-009-0052-1 19381459 PMC3139477

[B53] Choleva L. Janko K. (2013). Rise and persistence of animal polyploidy: evolutionary constraints and potential. Cytogenet. Genome Res. 140, 151–170 10.1159/000353464 23838539

[B54] Christ F. Debyser Z. (2013). The LEDGF/p75 integrase interaction, a novel target for anti-HIV therapy. Virology 435, 102–109 10.1016/j.virol.2012.09.033 23217620

[B55] Chung W. J. Okamura K. Martin R. Lai E. C. (2008). Endogenous RNA interference provides a somatic defense against Drosophila transposons. Curr. Biol. 18, 795–802 10.1016/j.cub.2008.05.006 18501606 PMC2812477

[B56] Ciuffi A. (2008). Mechanisms governing lentivirus integration site selection. Curr. Gene Ther. 8, 419–429 10.2174/156652308786848021 19075625

[B57] Collares-Pereira M. J. Matos I. Morgado-Santos M. Coelho M. M. (2013). Natural pathways towards polyploidy in animals: the *Squalius alburnoides* fish complex as a model system to study genome size and genome reorganization in polyploids. Cytogenet. Genome Res. 140, 97–116 10.1159/000351729 23796598

[B58] Conaway R. C. Conaway J. W. (2009). The INO80 chromatin remodeling complex in transcription, replication and repair. Trends Biochem. Sci. 34, 71–77 10.1016/j.tibs.2008.10.010 19062292

[B59] Conley A. B. Jordan I. K. (2012). Endogenous retroviruses and the epigenome, in Viruses: Essential Agents of Life, ed Witzany G. (Dordrecht: Spriinger), 309–323

[B60] Conley A. B. Piriyapongsa J. Jordan I. K. (2008). Retroviral promoters in the human genome. Bioinformatics 24, 1563–1567 10.1093/bioinformatics/btn243 18535086

[B61] Considine M. J. Wan Y. D'antuono M. F. Zhou Q. Han M. Gao H. (2012). Molecular genetic features of polyploidization and aneuploidization reveal unique patterns for genome duplication in diploid Malus. PLoS ONE 7:e29449 10.1371/journal.pone.0029449 22253724 PMC3254611

[B62] Consortium I. H. G. S. (2001). Initial sequencing and analysis of the human genome. Nature 409, 860–921 10.1038/35057062 11237011

[B63] Crews D. Gillette R. Scarpino S. V. Manikkam M. Savenkova M. I. Skinner M. K. (2012). Epigenetic transgenerational inheritance of altered stress responses. Proc. Natl. Acad. Sci. U.S.A. 109, 9143–9148 10.1073/pnas.1118514109 22615374 PMC3384163

[B64] Csorba T. Pantaleo V. Burgyan J. (2009). RNA silencing: an antiviral mechanism. Adv. Virus Res. 75, 35–71 10.1016/s0065-3527(09)07502-2 20109663

[B65] Czaja W. Mao P. Smerdon M. J. (2012). The emerging roles of ATP-dependent chromatin remodeling enzymes in nucleotide excision repair. Int. J. Mol. Sci. 13, 11954–11973 10.3390/ijms130911954 23109894 PMC3472786

[B66] Dai J. Xie W. Brady T. L. Gao J. Voytas D. F. (2007). Phosphorylation regulates integration of the yeast Ty5 retrotransposon into heterochromatin. Mol. Cell 27, 289–299 10.1016/j.molcel.2007.06.010 17643377

[B67] De Koning A. P. Gu W. Castoe T. A. Batzer M. A. Pollock D. D. (2011). Repetitive elements may comprise over two-thirds of the human genome. PLoS Genet. 7:e1002384 10.1371/journal.pgen.1002384 22144907 PMC3228813

[B68] De Rijck J. De Kogel C. Demeulemeester J. Vets S. El Ashkar S. Malani N. (2013). The BET family of proteins targets moloney murine leukemia virus integration near transcription start sites. Cell Rep. 5, 886–894 10.1016/j.celrep.2013.09.040 24183673 PMC4197836

[B69] Dickey J. S. Baird B. J. Redon C. E. Sokolov M. V. Sedelnikova O. A. Bonner W. M. (2009a). Intercellular communication of cellular stress monitored by gamma-H2AX induction. Carcinogenesis 30, 1686–1695 10.1093/carcin/bgp192 19651821 PMC2757548

[B70] Dickey J. S. Redon C. E. Nakamura A. J. Baird B. J. Sedelnikova O. A. Bonner W. M. (2009b). H2AX: functional roles and potential applications. Chromosoma 118, 683–692 10.1007/s00412-009-0234-4 19707781 PMC3094848

[B71] Dickey J. S. Zemp F. J. Altamirano A. Sedelnikova O. A. Bonner W. M. Kovalchuk O. (2011). H2AX phosphorylation in response to DNA double-strand break formation during bystander signalling: effect of microRNA knockdown. Radiat. Prot. Dosimetry 143, 264–269 10.1093/rpd/ncq470 21183548 PMC3108274

[B72] Ding D. Zhang L. Wang H. Liu Z. Zhang Z. Zheng Y. (2009). Differential expression of miRNAs in response to salt stress in maize roots. Ann. Bot. 103, 29–38 10.1093/aob/mcn205 18952624 PMC2707283

[B73] Ding S. W. Voinnet O. (2007). Antiviral immunity directed by small RNAs. Cell 130, 413–426 10.1016/j.cell.2007.07.039 17693253 PMC2703654

[B74] Ding S. Z. Goldberg J. B. Hatakeyama M. (2010). Helicobacter pylori infection, oncogenic pathways and epigenetic mechanisms in gastric carcinogenesis. Future Oncol. 6, 851–862 10.2217/fon.10.37 20465395 PMC2882595

[B75] Ding Y. Tao Y. Zhu C. (2013). Emerging roles of microRNAs in the mediation of drought stress response in plants. J. Exp. Bot. 64, 3077–3086 10.1093/jxb/ert164 23814278

[B76] Djordjevic M. Djordjevic M. Severinov K. (2012). CRISPR transcript processing: a mechanism for generating a large number of small interfering RNAs. Biol. Direct 7:24 10.1186/1745-6150-7-24 22849651 PMC3537551

[B77] Dowen R. H. Pelizzola M. Schmitz R. J. Lister R. Dowen J. M. Nery J. R. (2012). Widespread dynamic DNA methylation in response to biotic stress. Proc. Natl. Acad. Sci. U.S.A. 109, E2183–E2191 10.1073/pnas.1209329109 22733782 PMC3420206

[B78] Du P. Wu J. Zhang J. Zhao S. Zheng H. Gao G. (2011). Viral infection induces expression of novel phased microRNAs from conserved cellular microRNA precursors. PLoS Pathog. 7:e1002176 10.1371/journal.ppat.1002176 21901091 PMC3161970

[B79] Dumesic P. A. Madhani H. D. (2014). Recognizing the enemy within: licensing RNA-guided genome defense. Trends Biochem. Sci. 39, 25–34 10.1016/j.tibs.2013.10.003 24280023 PMC3902128

[B80] Dunn C. A. Van De Lagemaat L. N. Baillie G. J. Mager D. L. (2005). Endogenous retrovirus long terminal repeats as ready-to-use mobile promoters: the case of primate beta3GAL-T5. Gene 364, 2–12 10.1016/j.gene.2005.05.045 16112824

[B81] Ehya F. Monavarfeshani A. Mohseni Fard E. Karimi Farsad L. Khayam Nekouei M. Mardi M. (2013). Phytoplasma-responsive microRNAs modulate hormonal, nutritional, and stress signalling pathways in Mexican lime trees. PLoS ONE 8:e66372 10.1371/journal.pone.0066372 23824690 PMC3688891

[B82] Eissenberg J. C. (2012). Structural biology of the chromodomain: form and function. Gene 496, 69–78 10.1016/j.gene.2012.01.003 22285924

[B83] Eldem V. Celikkol Akcay U. Ozhuner E. Bakir Y. Uranbey S. Unver T. (2012). Genome-wide identification of miRNAs responsive to drought in peach (Prunus persica) by high-throughput deep sequencing. PLoS ONE 7:e50298 10.1371/journal.pone.0050298 23227166 PMC3515591

[B84] Erdel F. Krug J. Langst G. Rippe K. (2011). Targeting chromatin remodelers: signals and search mechanisms. Biochim. Biophys. Acta 1809, 497–508 10.1016/j.bbagrm.2011.06.005 21704204

[B85] Erdel F. Rippe K. (2011). Chromatin remodelling in mammalian cells by ISWI-type complexes–where, when and why? FEBS J. 278, 3608–3618 10.1111/j.1742-4658.2011.08282.x 21810179

[B86] Falbo K. B. Alabert C. Katou Y. Wu S. Han J. Wehr T. (2009). Involvement of a chromatin remodeling complex in damage tolerance during DNA replication. Nat. Struct. Mol. Biol. 16, 1167–1172 10.1038/nsmb.1686 19855395 PMC2974178

[B87] Fang L. Cheng F. Wu J. Wang X. (2012). The impact of genome triplication on tandem gene evolution in *Brassica rapa*. Front. Plant Sci. 3:261 10.3389/fpls.2012.00261 23226149 PMC3509317

[B88] Feldman M. Levy A. A. (2012). Genome evolution due to allopolyploidization in wheat. Genetics 192, 763–774 10.1534/genetics.112.146316 23135324 PMC3522158

[B89] Ferreira T. H. Gentile A. Vilela R. D. Costa G. G. Dias L. I. Endres L. (2012). microRNAs associated with drought response in the bioenergy crop sugarcane (Saccharum spp.). PLoS ONE 7:e46703 10.1371/journal.pone.0046703 23071617 PMC3469577

[B90] Feschotte C. (2008). Transposable elements and the evolution of regulatory networks. Nat. Rev. Genet. 9, 397–405 10.1038/nrg2337 18368054 PMC2596197

[B91] Firsanov D. V. Solovjeva L. V. Svetlova M. P. (2011). H2AX phosphorylation at the sites of DNA double-strand breaks in cultivated mammalian cells and tissues. Clin. Epigenetics 2, 283–297 10.1007/s13148-011-0044-4 22704343 PMC3365398

[B92] Franco S. Gostissa M. Zha S. Lombard D. B. Murphy M. M. Zarrin A. A. (2006). H2AX prevents DNA breaks from progressing to chromosome breaks and translocations. Mol. Cell 21, 201–214 10.1016/j.molcel.2006.01.005 16427010

[B93] Frias-Lasserre D. (2012). Non coding RNAs and viruses in the framework of the phylogeny of the genes, epigenesis and heredity. Int. J. Mol. Sci. 13, 477–490 10.3390/ijms13010477 22312265 PMC3269699

[B94] Fu B. Chen M. Zou M. Long M. He S. (2010). The rapid generation of chimerical genes expanding protein diversity in zebrafish. BMC Genomics 11:657 10.1186/1471-2164-11-657 21106061 PMC3091775

[B95] Fujimoto R. Kinoshita Y. Kawabe A. Kinoshita T. Takashima K. Nordborg M. (2008). Evolution and control of imprinted FWA genes in the genus Arabidopsis. PLoS Genet. 4:e1000048 10.1371/journal.pgen.1000048 18389059 PMC2270340

[B96] Fujioka M. Wu X. Jaynes J. B. (2009). A chromatin insulator mediates transgene homing and very long-range enhancer-promoter communication. Development 136, 3077–3087 10.1242/dev.036467 19675129 PMC2730365

[B97] Fukai E. Umehara Y. Sato S. Endo M. Kouchi H. Hayashi M. (2010). Derepression of the plant Chromovirus LORE1 induces germline transposition in regenerated plants. PLoS Genet. 6:e1000868 10.1371/journal.pgen.1000868 20221264 PMC2832683

[B98] Gangadharan S. Mularoni L. Fain-Thornton J. Wheelan S. J. Craig N. L. (2010). DNA transposon Hermes inserts into DNA in nucleosome-free regions *in vivo*. Proc. Natl. Acad. Sci. U.S.A. 107, 21966–21972 10.1073/pnas.1016382107 21131571 PMC3009821

[B99] Gao P. Bai X. Yang L. Lv D. Pan X. Li Y. (2011). osa-MIR393: a salinity- and alkaline stress-related microRNA gene. Mol. Biol. Rep. 38, 237–242 10.1007/s11033-010-0100-8 20336383

[B100] Gao X. Hou Y. Ebina H. Levin H. L. Voytas D. F. (2008). Chromodomains direct integration of retrotransposons to heterochromatin. Genome Res. 18, 359–369 10.1101/gr.7146408 18256242 PMC2259100

[B101] Garrett R. A. Vestergaard G. Shah S. A. (2011). Archaeal CRISPR-based immune systems: exchangeable functional modules. Trends Microbiol. 19, 549–556 10.1016/j.tim.2011.08.002 21945420

[B102] Gehring M. Bubb K. L. Henikoff S. (2009). Extensive demethylation of repetitive elements during seed development underlies gene imprinting. Science 324, 1447–1451 10.1126/science.1171609 19520961 PMC2886585

[B103] Gentile A. Ferreira T. H. Mattos R. S. Dias L. I. Hoshino A. A. Carneiro M. S. (2013). Effects of drought on the microtranscriptome of field-grown sugarcane plants. Planta 237, 783–798 10.1007/s00425-012-1795-7 23129215 PMC3579473

[B104] Gijsbers R. Ronen K. Vets S. Malani N. De Rijck J. Mcneely M. (2010). LEDGF hybrids efficiently retarget lentiviral integration into heterochromatin. Mol. Ther. 18, 552–560 10.1038/mt.2010.36 20195265 PMC2839429

[B105] Gorinsek B. Gubensek F. Kordis D. (2004). Evolutionary genomics of chromoviruses in eukaryotes. Mol. Biol. Evol. 21, 781–798 10.1093/molbev/msh057 14739248

[B106] Gospodinov A. Herceg Z. (2013). Shaping chromatin for repair. Mutat. Res. 752, 45–60 10.1016/j.mrrev.2012.10.001 23085398

[B107] Grandont L. Jenczewski E. Lloyd A. (2013). Meiosis and its deviations in polyploid plants. Cytogenet. Genome Res. 140, 171–184 10.1159/000351730 23817089

[B108] Greco M. Chiappetta A. Bruno L. Bitonti M. B. (2012). In *Posidonia oceanica* cadmium induces changes in DNA methylation and chromatin patterning. J. Exp. Bot. 63, 695–709 10.1093/jxb/err313 22058406 PMC3254685

[B109] Groszmann M. Greaves I. K. Albertyn Z. I. Scofield G. N. Peacock W. J. Dennis E. S. (2011). Changes in 24-nt siRNA levels in Arabidopsis hybrids suggest an epigenetic contribution to hybrid vigor. Proc. Natl. Acad. Sci. U.S.A. 108, 2617–2622 10.1073/pnas.1019217108 21266545 PMC3038704

[B110] Guetg C. Santoro R. (2012). Formation of nuclear heterochromatin: the nucleolar point of view. Epigenetics 7, 811–814 10.4161/epi.21072 22735386 PMC3427276

[B111] Guillou E. Ibarra A. Coulon V. Casado-Vela J. Rico D. Casal I. (2010). Cohesin organizes chromatin loops at DNA replication factories. Genes Dev. 24, 2812–2822 10.1101/gad.608210 21159821 PMC3003199

[B112] Guo X. Lu R. (2013). Characterization of virus-encoded RNA interference suppressors in *Caenorhabditis elegans*. J. Virol. 87, 5414–5423 10.1128/jvi.00148-13 23468484 PMC3648182

[B113] Gupta S. S. Maetzig T. Maertens G. N. Sharif A. Rothe M. Weidner-Glunde M. (2013). Bromo- and extraterminal domain chromatin regulators serve as cofactors for murine leukemia virus integration. J. Virol. 87, 12721–12736 10.1128/jvi.01942-13 24049186 PMC3838128

[B114] Hamon M. A. Cossart P. (2008). Histone modifications and chromatin remodeling during bacterial infections. Cell Host Microbe 4, 100–109 10.1016/j.chom.2008.07.009 18692770

[B115] Handler D. Meixner K. Pizka M. Lauss K. Schmied C. Gruber F. S. (2013). The genetic makeup of the Drosophila piRNA pathway. Mol. Cell 50, 762–777 10.1016/j.molcel.2013.04.031 23665231 PMC3679447

[B116] Hartley I. Elkhoury F. F. Heon Shin J. Xie B. Gu X. Gao Y. (2013). Long-lasting changes in DNA methylation following short-term hypoxic exposure in primary hippocampal neuronal cultures. PLoS ONE 8:e77859 10.1371/journal.pone.0077859 24205000 PMC3808424

[B117] He G. Chen B. Wang X. Li X. Li J. He H. (2013). Conservation and divergence of transcriptomic and epigenomic variation in maize hybrids. Genome Biol. 14:R57 10.1186/gb-2013-14-6-r57 23758703 PMC3707063

[B118] Hegarty M. Coate J. Sherman-Broyles S. Abbott R. Hiscock S. Doyle J. (2013). Lessons from natural and artificial polyploids in higher plants. Cytogenet. Genome Res. 140, 204–225 10.1159/000353361 23816545

[B119] Helmink B. A. Tubbs A. T. Dorsett Y. Bednarski J. J. Walker L. M. Feng Z. (2011). H2AX prevents CtIP-mediated DNA end resection and aberrant repair in G1-phase lymphocytes. Nature 469, 245–249 10.1038/nature09585 21160476 PMC3150591

[B120] Henikoff S. Dalal Y. (2005). Centromeric chromatin: what makes it unique? Curr. Opin. Genet. Dev. 15, 177–184 10.1016/j.gde.2005.01.004 15797200

[B121] Hizi A. Levin H. L. (2005). The integrase of the long terminal repeat-retrotransposon tf1 has a chromodomain that modulates integrase activities. J. Biol. Chem. 280, 39086–39094 10.1074/jbc.M506363200 16188891

[B122] Holeski L. M. Jander G. Agrawal A. A. (2012). Transgenerational defense induction and epigenetic inheritance in plants. Trends Ecol. Evol. 27, 618–626 10.1016/j.tree.2012.07.011 22940222

[B123] Hossain M. B. Vahter M. Concha G. Broberg K. (2012). Low-level environmental cadmium exposure is associated with DNA hypomethylation in Argentinean women. Environ. Health Perspect. 120, 879–884 10.1289/ehp.1104600 22382075 PMC3385444

[B124] Huang H. Jiao R. (2012). Roles of chromatin assembly factor 1 in the epigenetic control of chromatin plasticity. Sci. China Life Sci. 55, 15–19 10.1007/s11427-012-4269-z 22314486

[B125] Huang T. Zhang X. (2013). Host defense against DNA virus infection in shrimp is mediated by the siRNA pathway. Eur. J. Immunol. 43, 137–146 10.1002/eji.201242806 23065729

[B126] Hunt C. R. Ramnarain D. Horikoshi N. Iyengar P. Pandita R. K. Shay J. W. (2013). Histone modifications and DNA double-strand break repair after exposure to ionizing radiations. Radiat. Res. 179, 383–392 10.1667/rr3308.2 23373901 PMC4133051

[B127] Inacio A. Pinho J. Pereira P. M. Comai L. Coelho M. M. (2012). Global analysis of the small RNA transcriptome in different ploidies and genomic combinations of a vertebrate complex–the *Squalius alburnoides*. PLoS ONE 7:e41158 10.1371/journal.pone.0041158 22815952 PMC3399795

[B128] Iyer N. J. Jia X. Sunkar R. Tang G. Mahalingam R. (2012). microRNAs responsive to ozone-induced oxidative stress in *Arabidopsis thaliana*. Plant Signal. Behav. 7, 484–491 10.4161/psb.19337 22499183 PMC3419038

[B129] Jacobsen S. C. Brons C. Bork-Jensen J. Ribel-Madsen R. Yang B. Lara E. (2012). Effects of short-term high-fat overfeeding on genome-wide DNA methylation in the skeletal muscle of healthy young men. Diabetologia 55, 3341–3349 10.1007/s00125-012-2717-8 22961225

[B130] Jiang L. Wei C. Li Y. (2012). Viral suppression of RNA silencing. Sci. China Life Sci. 55, 109–118 10.1007/s11427-012-4279-x 22415681

[B131] Kadyrova L. Y. Mertz T. M. Zhang Y. Northam M. R. Sheng Z. Lobachev K. S. (2013). A reversible histone h3 acetylation cooperates with mismatch repair and replicative polymerases in maintaining genome stability. PLoS Genet. 9:e1003899 10.1371/journal.pgen.1003899 24204308 PMC3812082

[B132] Kalmykova A. I. Klenov M. S. Gvozdev V. A. (2005). Argonaute protein PIWI controls mobilization of retrotransposons in the Drosophila male germline. Nucleic Acids Res. 33, 2052–2059 10.1093/nar/gki323 15817569 PMC1074743

[B133] Kaneda A. Matsusaka K. Aburatani H. Fukayama M. (2012). Epstein-Barr virus infection as an epigenetic driver of tumorigenesis. Cancer Res. 72, 3445–3450 10.1158/0008-5472.can-11-3919 22761333

[B134] Kapusta A. Kronenberg Z. Lynch V. J. Zhuo X. Ramsay L. Bourque G. (2013). Transposable elements are major contributors to the origin, diversification, and regulation of vertebrate long noncoding RNAs. PLoS Genet. 9:e1003470 10.1371/journal.pgen.1003470 23637635 PMC3636048

[B135] Karan R. Deleon T. Biradar H. Subudhi P. K. (2012). Salt stress induced variation in DNA methylation pattern and its influence on gene expression in contrasting rice genotypes. PLoS ONE 7:e40203 10.1371/journal.pone.0040203 22761959 PMC3386172

[B136] Kassis J. A. (2002). Pairing-sensitive silencing, polycomb group response elements, and transposon homing in Drosophila. Adv. Genet. 46, 421–438 10.1016/S0065-2660(02)46015-4 11931233

[B137] Kathiria P. Sidler C. Golubov A. Kalischuk M. Kawchuk L. M. Kovalchuk I. (2010). Tobacco mosaic virus infection results in an increase in recombination frequency and resistance to viral, bacterial, and fungal pathogens in the progeny of infected tobacco plants. Plant Physiol. 153, 1859–1870 10.1104/pp.110.157263 20498336 PMC2923882

[B138] Kawamura Y. Saito K. Kin T. Ono Y. Asai K. Sunohara T. (2008). Drosophila endogenous small RNAs bind to Argonaute 2 in somatic cells. Nature 453, 793–797 10.1038/nature06938 18463636

[B139] Kenan-Eichler M. Leshkowitz D. Tal L. Noor E. Melamed-Bessudo C. Feldman M. (2011). Wheat hybridization and polyploidization results in deregulation of small RNAs. Genetics 188, 263–272 10.1534/genetics.111.128348 21467573 PMC3122319

[B140] Kettles G. J. Drurey C. Schoonbeek H. J. Maule A. J. Hogenhout S. A. (2013). Resistance of *Arabidopsis thaliana* to the green peach aphid, *Myzus persicae*, involves camalexin and is regulated by microRNAs. New Phytol. 198, 1178–1190 10.1111/nph.12218 23528052 PMC3666093

[B141] Khraiwesh B. Zhu J. K. Zhu J. (2012). Role of miRNAs and siRNAs in biotic and abiotic stress responses of plants. Biochim. Biophys. Acta 1819, 137–148 10.1016/j.bbagrm.2011.05.001 21605713 PMC3175014

[B142] Kinner A. Wu W. Staudt C. Iliakis G. (2008). Gamma-H2AX in recognition and signaling of DNA double-strand breaks in the context of chromatin. Nucleic Acids Res. 36, 5678–5694 10.1093/nar/gkn550 18772227 PMC2553572

[B143] Kinoshita Y. Saze H. Kinoshita T. Miura A. Soppe W. J. Koornneef M. (2007). Control of FWA gene silencing in *Arabidopsis thaliana* by SINE-related direct repeats. Plant J. 49, 38–45 10.1111/j.1365-313X.2006.02936.x 17144899

[B144] Kokosar J. Kordis D. (2013). Genesis and regulatory wiring of retroelement-derived domesticated genes: a phylogenomic perspective. Mol. Biol. Evol. 30, 1015–1031 10.1093/molbev/mst014 23348003 PMC3670739

[B145] Kordis D. (2005). A genomic perspective on the chromodomain-containing retrotransposons: chromoviruses. Gene 347, 161–173 10.1016/j.gene.2004.12.017 15777633

[B146] Korzeniewski N. Spardy N. Duensing A. Duensing S. (2011). Genomic instability and cancer: lessons learned from human papillomaviruses. Cancer Lett. 305, 113–122 10.1016/j.canlet.2010.10.013 21075512 PMC3046211

[B147] Kou H. P. Li Y. Song X. X. Ou X. F. Xing S. C. Ma J. (2011). Heritable alteration in DNA methylation induced by nitrogen-deficiency stress accompanies enhanced tolerance by progenies to the stress in rice (*Oryza sativa* L.). J. Plant Physiol. 168, 1685–1693 10.1016/j.jplph.2011.03.017 21665325

[B148] Kraitshtein Z. Yaakov B. Khasdan V. Kashkush K. (2010). Genetic and epigenetic dynamics of a retrotransposon after allopolyploidization of wheat. Genetics 186, 801–812 10.1534/genetics.110.120790 20823338 PMC2975285

[B149] Kulcheski F. R. De Oliveira L. F. Molina L. G. Almerao M. P. Rodrigues F. A. Marcolino J. (2011). Identification of novel soybean microRNAs involved in abiotic and biotic stresses. BMC Genomics 12:307 10.1186/1471-2164-12-307 21663675 PMC3141666

[B150] Labonte B. Suderman M. Maussion G. Navaro L. Yerko V. Mahar I. (2012). Genome-wide epigenetic regulation by early-life trauma. Arch. Gen. Psychiatry 69, 722–731 10.1001/archgenpsychiatry.2011.2287 22752237 PMC4991944

[B151] Lahue R. S. Frizzell A. (2012). Histone deacetylase complexes as caretakers of genome stability. Epigenetics 7, 806–810 10.4161/epi.20922 22722985 PMC3427275

[B152] Law J. A. Jacobsen S. E. (2010). Establishing, maintaining and modifying DNA methylation patterns in plants and animals. Nat. Rev. Genet. 11, 204–220 10.1038/nrg2719 20142834 PMC3034103

[B153] Lee H. C. Gu W. Shirayama M. Youngman E. Conte D. Jr. Mello C. C. (2012). *C. elegans* piRNAs mediate the genome-wide surveillance of germline transcripts. Cell 150, 78–87 10.1016/j.cell.2012.06.016 22738724 PMC3410639

[B154] Lelandais-Briere C. Sorin C. Crespi M. Hartmann C. (2012). [Non-coding RNAs involved in plant responses to environmental constraints]. Biol. Aujourdhui. 206, 313–322 10.1051/jbio/2012032 23419258

[B155] Leonard S. Wei W. Anderton J. Vockerodt M. Rowe M. Murray P. G. (2011). Epigenetic and transcriptional changes which follow Epstein-Barr virus infection of germinal center B cells and their relevance to the pathogenesis of Hodgkin's lymphoma. J. Virol. 85, 9568–9577 10.1128/jvi.00468-11 21752916 PMC3165764

[B156] Lewinski M. K. Yamashita M. Emerman M. Ciuffi A. Marshall H. Crawford G. (2006). Retroviral DNA integration: viral and cellular determinants of target-site selection. PLoS Pathog. 2:e60 10.1371/journal.ppat.0020060 16789841 PMC1480600

[B157] Li B. Duan H. Li J. Deng X. W. Yin W. Xia X. (2013). Global identification of miRNAs and targets in *Populus euphratica* under salt stress. Plant Mol. Biol. 81, 525–539 10.1007/s11103-013-0010-y 23430564

[B158] Li B. Qin Y. Duan H. Yin W. Xia X. (2011a). Genome-wide characterization of new and drought stress responsive microRNAs in *Populus euphratica*. J. Exp. Bot. 62, 3765–3779 10.1093/jxb/err051 21511902 PMC3134338

[B159] Li H. Dong Y. Yin H. Wang N. Yang J. Liu X. (2011b). Characterization of the stress associated microRNAs in Glycine max by deep sequencing. BMC Plant Biol. 11:170 10.1186/1471-2229-11-170 22112171 PMC3267681

[B160] Li Y. Lu Y. G. Shi Y. Wu L. Xu Y. J. Huang F. (2014). Multiple rice microRNAs are involved in immunity against the blast fungus *Magnaporthe oryzae*. Plant Physiol. 164, 1077–1092 10.1104/pp.113.230052 24335508 PMC3912081

[B161] Lim J. P. Brunet A. (2013). Bridging the transgenerational gap with epigenetic memory. Trends Genet. 29, 176–186 10.1016/j.tig.2012.12.008 23410786 PMC3595609

[B162] Lim K. Y. Soltis D. E. Soltis P. S. Tate J. Matyasek R. Srubarova H. (2008). Rapid chromosome evolution in recently formed polyploids in Tragopogon (Asteraceae). PLoS ONE 3:e3353 10.1371/journal.pone.0003353 18843372 PMC2556386

[B163] Lindblad-Toh K. Garber M. Zuk O. Lin M. F. Parker B. J. Washietl S. (2011). A high-resolution map of human evolutionary constraint using 29 mammals. Nature 478, 476–482 10.1038/nature10530 21993624 PMC3207357

[B164] Linger J. G. Tyler J. K. (2007). Chromatin disassembly and reassembly during DNA repair. Mutat. Res. 618, 52–64 10.1016/j.mrfmmm.2006.05.039 17303193 PMC2593076

[B165] Lisby M. Rothstein R. (2005). Localization of checkpoint and repair proteins in eukaryotes. Biochimie 87, 579–589 10.1016/j.biochi.2004.10.023 15989975

[B166] Liu Q. Gong Z. (2011). The coupling of epigenome replication with DNA replication. Curr. Opin. Plant Biol. 14, 187–194 10.1016/j.pbi.2010.12.001 21233006

[B167] Liu Z. Kumari S. Zhang L. Zheng Y. Ware D. (2012). Characterization of miRNAs in response to short-term waterlogging in three inbred lines of Zea mays. PLoS ONE 7:e39786 10.1371/journal.pone.0039786 22768123 PMC3387268

[B168] Llano M. Saenz D. T. Meehan A. Wongthida P. Peretz M. Walker W. H. (2006). An essential role for LEDGF/p75 in HIV integration. Science 314, 461–464 10.1126/science.1132319 16959972

[B169] Long M. (2001). Evolution of novel genes. Curr. Opin. Genet. Dev. 11, 673–680 10.1016/S0959-437X(00)00252-5 11682312

[B170] Lowe C. B. Kellis M. Siepel A. Raney B. J. Clamp M. Salama S. R. (2011). Three periods of regulatory innovation during vertebrate evolution. Science 333, 1019–1024 10.1126/science.1202702 21852499 PMC3511857

[B171] Luna E. Ton J. (2012). The epigenetic machinery controlling transgenerational systemic acquired resistance. Plant Signal. Behav. 7, 615–618 10.4161/psb.20155 22580690 PMC3442853

[B172] Luo J. Hao M. Zhang L. Chen J. Zhang L. Yuan Z. (2012). Microsatellite mutation rate during allohexaploidization of newly resynthesized wheat. Int. J. Mol. Sci. 13, 12533–12543 10.3390/ijms131012533 23202911 PMC3497285

[B173] Macovei A. Tuteja N. (2012). microRNAs targeting DEAD-box helicases are involved in salinity stress response in rice (*Oryza sativa* L.). BMC Plant Biol. 12:183 10.1186/1471-2229-12-183 23043463 PMC3502329

[B174] Madlung A. Wendel J. F. (2013). Genetic and epigenetic aspects of polyploid evolution in plants. Cytogenet. Genome Res. 140, 270–285 10.1159/000351430 23751292

[B175] Maillard P. V. Ciaudo C. Marchais A. Li Y. Jay F. Ding S. W. (2013). Antiviral RNA interference in mammalian cells. Science 342, 235–238 10.1126/science.1241930 24115438 PMC3853215

[B176] Maksakova I. A. Romanish M. T. Gagnier L. Dunn C. A. Van De Lagemaat L. N. Mager D. L. (2006). Retroviral elements and their hosts: insertional mutagenesis in the mouse germ line. PLoS Genet. 2:e2 10.1371/journal.pgen.0020002 16440055 PMC1331978

[B177] Manikkam M. Tracey R. Guerrero-Bosagna C. Skinner M. K. (2012). Dioxin (TCDD) induces epigenetic transgenerational inheritance of adult onset disease and sperm epimutations. PLoS ONE 7:e46249 10.1371/journal.pone.0046249 23049995 PMC3458876

[B178] Manikkam M. Tracey R. Guerrero-Bosagna C. Skinner M. K. (2013). Plastics derived endocrine disruptors (BPA, DEHP and DBP) induce epigenetic transgenerational inheritance of obesity, reproductive disease and sperm epimutations. PLoS ONE 8:e55387 10.1371/journal.pone.0055387 23359474 PMC3554682

[B179] Marraffini L. A. Sontheimer E. J. (2010). CRISPR interference: RNA-directed adaptive immunity in bacteria and archaea. Nat. Rev. Genet. 11, 181–190 10.1038/nrg2749 20125085 PMC2928866

[B180] Martis M. M. Zhou R. Haseneyer G. Schmutzer T. Vrana J. Kubalakova M. (2013). Reticulate evolution of the rye genome. Plant Cell 25, 3685–3698 10.1105/tpc.113.114553 24104565 PMC3877785

[B181] Masaki T. Qu J. Cholewa-Waclaw J. Burr K. Raaum R. Rambukkana A. (2013). Reprogramming adult Schwann cells to stem cell-like cells by leprosy bacilli promotes dissemination of infection. Cell 152, 51–67 10.1016/j.cell.2012.12.014 23332746 PMC4314110

[B182] Matsunaga W. Kobayashi A. Kato A. Ito H. (2012). The effects of heat induction and the siRNA biogenesis pathway on the transgenerational transposition of ONSEN, a copia-like retrotransposon in *Arabidopsis thaliana*. Plant Cell Physiol. 53, 824–833 10.1093/pcp/pcr179 22173101

[B183] Mccue A. D. Nuthikattu S. Reeder S. H. Slotkin R. K. (2012). Gene expression and stress response mediated by the epigenetic regulation of a transposable element small RNA. PLoS Genet. 8:e1002474 10.1371/journal.pgen.1002474 22346759 PMC3276544

[B184] Meehan A. M. Poeschla E. M. (2010). Chromatin tethering and retroviral integration: recent discoveries and parallels with DNA viruses. Biochim. Biophys. Acta 1799, 182–191 10.1016/j.bbagrm.2009.10.001 19836475 PMC7326329

[B185] Mercer T. R. Edwards S. L. Clark M. B. Neph S. J. Wang H. Stergachis A. B. (2013). DNase I-hypersensitive exons colocalize with promoters and distal regulatory elements. Nat. Genet. 45, 852–859 10.1038/ng.2677 23793028 PMC4405174

[B186] Mercer T. R. Mattick J. S. (2013). Understanding the regulatory and transcriptional complexity of the genome through structure. Genome Res. 23, 1081–1088 10.1101/gr.156612.113 23817049 PMC3698501

[B187] Mermoud J. E. Rowbotham S. P. Varga-Weisz P. D. (2011). Keeping chromatin quiet: how nucleosome remodeling restores heterochromatin after replication. Cell Cycle 10, 4017–4025 10.4161/cc.10.23.18558 22101266 PMC3272284

[B188] Michalak M. Barciszewska M. Z. Barciszewski J. Plitta B. P. Chmielarz P. (2013). Global changes in DNA methylation in seeds and seedlings of *Pyrus communis* after seed desiccation and storage. PLoS ONE 8:e70693 10.1371/journal.pone.0070693 23940629 PMC3734228

[B189] Miller K. M. Tjeertes J. V. Coates J. Legube G. Polo S. E. Britton S. (2010). Human HDAC1 and HDAC2 function in the DNA-damage response to promote DNA nonhomologous end-joining. Nat. Struct. Mol. Biol. 17, 1144–1151 10.1038/nsmb.1899 20802485 PMC3018776

[B190] Miller M. Zhang C. Chen Z. J. (2012). Ploidy and hybridity effects on growth vigor and gene expression in *Arabidopsis thaliana* hybrids and their parents. G3 (Bethesda) 2, 505–513 10.1534/g3.112.002162 22540042 PMC3337479

[B191] Mirouze M. Reinders J. Bucher E. Nishimura T. Schneeberger K. Ossowski S. (2009). Selective epigenetic control of retrotransposition in Arabidopsis. Nature 461, 427–430 10.1038/nature08328 19734882

[B192] Moldovan D. Spriggs A. Yang J. Pogson B. J. Dennis E. S. Wilson I. W. (2010). Hypoxia-responsive microRNAs and trans-acting small interfering RNAs in Arabidopsis. J. Exp. Bot. 61, 165–177 10.1093/jxb/erp296 19815687 PMC2791121

[B193] Mou Z. Kenny A. E. Curcio M. J. (2006). Hos2 and Set3 promote integration of Ty1 retrotransposons at tRNA genes in *Saccharomyces cerevisiae*. Genetics 172, 2157–2167 10.1534/genetics.105.054072 16415356 PMC1456361

[B194] Mularoni L. Zhou Y. Bowen T. Gangadharan S. Wheelan S. J. Boeke J. D. (2012). Retrotransposon Ty1 integration targets specifically positioned asymmetric nucleosomal DNA segments in tRNA hotspots. Genome Res. 22, 693–703 10.1101/gr.129460.111 22219510 PMC3317151

[B195] Munoz-Galvan S. Jimeno S. Rothstein R. Aguilera A. (2013). Histone H3K56 acetylation, Rad52, and non-DNA repair factors control double-strand break repair choice with the sister chromatid. PLoS Genet. 9:e1003237 10.1371/journal.pgen.1003237 23357952 PMC3554610

[B196] Nakaminami K. Matsui A. Shinozaki K. Seki M. (2012). RNA regulation in plant abiotic stress responses. Biochim. Biophys. Acta 1819, 149–153 10.1016/j.bbagrm.2011.07.015 21840431

[B197] Nakayashiki H. (2011). The Trickster in the genome: contribution and control of transposable elements. Genes Cells 16, 827–841 10.1111/j.1365-2443.2011.01533.x 21722269

[B198] Narlikar G. J. Sundaramoorthy R. Owen-Hughes T. (2013). Mechanisms and functions of ATP-dependent chromatin-remodeling enzymes. Cell 154, 490–503 10.1016/j.cell.2013.07.011 23911317 PMC3781322

[B199] Navarro B. Pantaleo V. Gisel A. Moxon S. Dalmay T. Bisztray G. (2009). Deep sequencing of viroid-derived small RNAs from grapevine provides new insights on the role of RNA silencing in plant-viroid interaction. PLoS ONE 4:e7686 10.1371/journal.pone.0007686 19890399 PMC2767511

[B200] Neumann P. Navratilova A. Koblizkova A. Kejnovsky E. Hribova E. Hobza R. (2011). Plant centromeric retrotransposons: a structural and cytogenetic perspective. Mob. DNA 2:4 10.1186/1759-8753-2-4 21371312 PMC3059260

[B201] Neumann P. Yan H. Jiang J. (2007). The centromeric retrotransposons of rice are transcribed and differentially processed by RNA interference. Genetics 176, 749–761 10.1534/genetics.107.071902 17409063 PMC1894605

[B202] Neves-Costa A. Varga-Weisz P. (2006). The roles of chromatin remodelling factors in replication. Results Probl. Cell Differ. 41, 91–107 10.1007/400_007 16909892

[B203] Ng D. W. Lu J. Chen Z. J. (2012). Big roles for small RNAs in polyploidy, hybrid vigor, and hybrid incompatibility. Curr. Opin. Plant Biol. 15, 154–161 10.1016/j.pbi.2012.01.007 22326630

[B204] Novikov A. Smyshlyaev G. Novikova O. (2012). Evolutionary history of LTR retrotransposon chromodomains in plants. Int. J. Plant Genomics 2012:874743 10.1155/2012/874743 22611377 PMC3350952

[B205] Nuthikattu S. Mccue A. D. Panda K. Fultz D. Defraia C. Thomas E. N. (2013). The initiation of epigenetic silencing of active transposable elements is triggered by RDR6 and 21-22 nucleotide small interfering RNAs. Plant Physiol. 162, 116–131 10.1104/pp.113.216481 23542151 PMC3641197

[B206] Omarov R. T. Scholthof H. B. (2012). Biological chemistry of virus-encoded suppressors of RNA silencing: an overview. Methods Mol. Biol. 894, 39–56 10.1007/978-1-61779-882-5_3 22678571

[B207] Osley M. A. Tsukuda T. Nickoloff J. A. (2007). ATP-dependent chromatin remodeling factors and DNA damage repair. Mutat. Res. 618, 65–80 10.1016/j.mrfmmm.2006.07.011 17291544 PMC1904433

[B208] Ou X. Zhang Y. Xu C. Lin X. Zang Q. Zhuang T. (2012). Transgenerational inheritance of modified DNA methylation patterns and enhanced tolerance induced by heavy metal stress in rice (*Oryza sativa* L.). PLoS ONE 7:e41143 10.1371/journal.pone.0041143 22984395 PMC3439459

[B209] Ozhuner E. Eldem V. Ipek A. Okay S. Sakcali S. Zhang B. (2013). Boron stress responsive microRNAs and their targets in barley. PLoS ONE 8:e59543 10.1371/journal.pone.0059543 23555702 PMC3608689

[B210] Palomera-Sanchez Z. Zurita M. (2011). Open, repair and close again: chromatin dynamics and the response to UV-induced DNA damage. DNA Repair (Amst.) 10, 119–125 10.1016/j.dnarep.2010.10.010 21130713

[B211] Pang M. Woodward A. W. Agarwal V. Guan X. Ha M. Ramachandran V. (2009). Genome-wide analysis reveals rapid and dynamic changes in miRNA and siRNA sequence and expression during ovule and fiber development in allotetraploid cotton (*Gossypium hirsutum* L.). Genome Biol. 10:R122 10.1186/gb-2009-10-11-r122 19889219 PMC3091316

[B212] Pantaleo V. (2011). Plant RNA silencing in viral defence. Adv. Exp. Med. Biol. 722, 39–58 10.1007/978-1-4614-0332-6_3 21915781

[B213] Partridge J. F. (2008). Centromeric chromatin in fission yeast. Front. Biosci. 13, 3896–3905 10.2741/2977 18508483

[B214] Pask A. J. Papenfuss A. T. Ager E. I. Mccoll K. A. Speed T. P. Renfree M. B. (2009). Analysis of the platypus genome suggests a transposon origin for mammalian imprinting. Genome Biol. 10:R1 10.1186/gb-2009-10-1-r1 19121219 PMC2687786

[B215] Peaston A. E. Evsikov A. V. Graber J. H. De Vries W. N. Holbrook A. E. Solter D. (2004). Retrotransposons regulate host genes in mouse oocytes and preimplantation embryos. Dev. Cell 7, 597–606 10.1016/j.devcel.2004.09.004 15469847

[B216] Pelaez P. Sanchez F. (2013). Small RNAs in plant defense responses during viral and bacterial interactions: similarities and differences. Front. Plant Sci. 4:343 10.3389/fpls.2013.00343 24046772 PMC3763480

[B217] Perez-Quintero A. L. Quintero A. Urrego O. Vanegas P. Lopez C. (2012). Bioinformatic identification of cassava miRNAs differentially expressed in response to infection by *Xanthomonas axonopodis* pv. manihotis. BMC Plant Biol. 12:29 10.1186/1471-2229-12-29 22361011 PMC3337288

[B218] Plate I. Hallwyl S. C. Shi I. Krejci L. Muller C. Albertsen L. (2008). Interaction with RPA is necessary for Rad52 repair center formation and for its mediator activity. J. Biol. Chem. 283, 29077–29085 10.1074/jbc.M804881200 18703507 PMC2570898

[B219] Poot R. A. Bozhenok L. Van Den Berg D. L. Hawkes N. Varga-Weisz P. D. (2005). Chromatin remodeling by WSTF-ISWI at the replication site: opening a window of opportunity for epigenetic inheritance? Cell Cycle 4, 543–546 10.4161/cc.4.4.1624 15753658

[B220] Price B. D. D'andrea A. D. (2013). Chromatin remodeling at DNA double-strand breaks. Cell 152, 1344–1354 10.1016/j.cell.2013.02.011 23498941 PMC3670600

[B221] Qin Y. Duan Z. Xia X. Yin W. (2011). Expression profiles of precursor and mature microRNAs under dehydration and high salinity shock in *Populus euphratica*. Plant Cell Rep. 30, 1893–1907 10.1007/s00299-011-1096-9 21706230

[B222] Queen K. J. Shi M. Zhang F. Cvek U. Scott R. S. (2013). Epstein-Barr virus-induced epigenetic alterations following transient infection. Int. J. Cancer 132, 2076–2086 10.1002/ijc.27893 23047626 PMC3578144

[B223] Quintero A. Perez-Quintero A. L. Lopez C. (2013). Identification of ta-siRNAs and cis-nat-siRNAs in cassava and their roles in response to cassava bacterial blight. Genom. Proteomics Bioinform. 11, 172–181 10.1016/j.gpb.2013.03.001 23665476 PMC4357781

[B224] Rahavi M. R. Migicovsky Z. Titov V. Kovalchuk I. (2011). Transgenerational adaptation to heavy metal salts in Arabidopsis. Front. Plant Sci. 2:91 10.3389/fpls.2011.00091 22639617 PMC3355606

[B225] Raja P. Jackel J. N. Li S. Heard I. M. Bisaro D. M. (2014). Arabidopsis double-stranded RNA binding protein DRB3 participates in methylation-mediated defense against geminiviruses. J. Virol. 88, 2611–2622 10.1128/JVI.02305-13 24352449 PMC3958096

[B226] Raman V. Simon S. A. Romag A. Demirci F. Mathioni S. M. Zhai J. (2013). Physiological stressors and invasive plant infections alter the small RNA transcriptome of the rice blast fungus, Magnaporthe oryzae. BMC Genomics 14:326 10.1186/1471-2164-14-326 23663523 PMC3658920

[B227] Ramesh S. V. Ratnaparkhe M. B. Kumawat G. Gupta G. K. Husain S. M. (2014). Plant miRNAome and antiviral resistance: a retrospective view and prospective challenges. Virus Genes. 48, 1–14 10.1007/s11262-014-1038-z 24445902

[B228] Rechavi O. Minevich G. Hobert O. (2011). Transgenerational inheritance of an acquired small RNA-based antiviral response in *C. elegans*. Cell 147, 1248–1256 10.1016/j.cell.2011.10.042 22119442 PMC3250924

[B229] Redon C. E. Dickey J. S. Bonner W. M. Sedelnikova O. A. (2009). gamma-H2AX as a biomarker of DNA damage induced by ionizing radiation in human peripheral blood lymphocytes and artificial skin. Adv. Space Res. 43, 1171–1178 10.1016/j.asr.2008.10.011 20046946 PMC2735274

[B230] Ren Y. Chen L. Zhang Y. Kang X. Zhang Z. Wang Y. (2012). Identification of novel and conserved Populus tomentosa microRNA as components of a response to water stress. Funct. Integr. Genomics 12, 327–339 10.1007/s10142-012-0271-6 22415631

[B231] Ren Y. Chen L. Zhang Y. Kang X. Zhang Z. Wang Y. (2013). Identification and characterization of salt-responsive microRNAs in Populus tomentosa by high-throughput sequencing. Biochimie 95, 743–750 10.1016/j.biochi.2012.10.025 23142627

[B232] Rigal M. Mathieu O. (2011). A “mille-feuille” of silencing: epigenetic control of transposable elements. Biochim. Biophys. Acta 1809, 452–458 10.1016/j.bbagrm.2011.04.001 21514406

[B233] Robert T. Vanoli F. Chiolo I. Shubassi G. Bernstein K. A. Rothstein R. (2011). HDACs link the DNA damage response, processing of double-strand breaks and autophagy. Nature 471, 74–79 10.1038/nature09803 21368826 PMC3935290

[B234] Rogakou E. P. Pilch D. R. Orr A. H. Ivanova V. S. Bonner W. M. (1998). DNA double-stranded breaks induce histone H2AX phosphorylation on serine 139. J. Biol. Chem. 273, 5858–5868 9488723 10.1074/jbc.273.10.5858

[B235] Rongrui L. Na H. Zongfang L. Fanpu J. Shiwen J. (2014). Epigenetic mechanism involved in the HBV/HCV-related hepatocellular carcinoma tumorigenesis. Curr. Pharm. Des. 20, 1715–1725 10.2174/13816128113199990533 23888939

[B236] Ruiz-Ferrer V. Voinnet O. (2009). Roles of plant small RNAs in biotic stress responses. Annu. Rev. Plant Biol. 60, 485–510 10.1146/annurev.arplant.043008.092111 19519217

[B237] Ryan D. P. Owen-Hughes T. (2011). Snf2-family proteins: chromatin remodellers for any occasion. Curr. Opin. Chem. Biol. 15, 649–656 10.1016/j.cbpa.2011.07.022 21862382 PMC4162295

[B238] Sabin L. R. Cherry S. (2013). Small creatures use small RNAs to direct antiviral defenses. Eur. J. Immunol. 43, 27–33 10.1002/eji.201243201 23322691 PMC3799994

[B239] Sani E. Herzyk P. Perrella G. Colot V. Amtmann A. (2013). Hyperosmotic priming of Arabidopsis seedlings establishes a long-term somatic memory accompanied by specific changes of the epigenome. Genome Biol. 14:R59 10.1186/gb-2013-14-6-r59 23767915 PMC3707022

[B240] Savva Y. A. Jepson J. E. Chang Y. J. Whitaker R. Jones B. C. St. Laurent G. (2013). RNA editing regulates transposon-mediated heterochromatic gene silencing. Nat. Commun. 4:2745 10.1038/ncomms3745 24201902 PMC3992701

[B241] Schmidt C. K. Jackson S. P. (2013). On your mark, get SET(D2), go! H3K36me3 primes DNA mismatch repair. Cell 153, 513–515 10.1016/j.cell.2013.04.018 23622237

[B242] Seeber A. Hauer M. Gasser S. M. (2013). Nucleosome remodelers in double-strand break repair. Curr. Opin. Genet. Dev. 23, 174–184 10.1016/j.gde.2012.12.008 23352131

[B243] Sha A. Zhao J. Yin K. Tang Y. Wang Y. Wei X. (2014). Virus-based microRNA silencing in plants. Plant Physiol. 164, 36–47 10.1104/pp.113.231100 24296072 PMC3875814

[B244] Shapiro J. A. (2011). Evolution: a View from the 21st Century. Upper Saddle River, NJ: FT Press Science

[B339] Shapiro J. A. (2014). The physiology of the Read-Write (RW) genome. J. Physiol. (in press). 10.1016/j.plrev.2013.07.016 24882816 PMC4048091

[B245] Sharma A. (2013). Transgenerational epigenetic inheritance: focus on soma to germline information transfer. Prog. Biophys. Mol. Biol. 113, 439–446 10.1016/j.pbiomolbio.2012.12.003 23257323

[B246] Sharma A. Larue R. C. Plumb M. R. Malani N. Male F. Slaughter A. (2013a). BET proteins promote efficient murine leukemia virus integration at transcription start sites. Proc. Natl. Acad. Sci. U.S.A. 110, 12036–12041 10.1073/pnas.1307157110 23818621 PMC3718171

[B247] Sharma A. Wolfgruber T. K. Presting G. G. (2013b). Tandem repeats derived from centromeric retrotransposons. BMC Genomics 14:142 10.1186/1471-2164-14-142 23452340 PMC3648361

[B248] Shi L. Oberdoerffer P. (2012). Chromatin dynamics in DNA double-strand break repair. Biochim. Biophys. Acta 1819, 811–819 10.1016/j.bbagrm.2012.01.002 22285574 PMC3368990

[B249] Shivaprasad P. V. Dunn R. M. Santos B. A. Bassett A. Baulcombe D. C. (2012). Extraordinary transgressive phenotypes of hybrid tomato are influenced by epigenetics and small silencing RNAs. EMBO J. 31, 257–266 10.1038/emboj.2011.458 22179699 PMC3261569

[B250] Shpiz S. Kwon D. Rozovsky Y. Kalmykova A. (2009). rasiRNA pathway controls antisense expression of Drosophila telomeric retrotransposons in the nucleus. Nucleic Acids Res. 37, 268–278 10.1093/nar/gkn960 19036789 PMC2615633

[B251] Shu L. Hu Z. (2012). Characterization and differential expression of microRNAs elicited by sulfur deprivation in *Chlamydomonas reinhardtii*. BMC Genomics 13:108 10.1186/1471-2164-13-108 22439676 PMC3441669

[B252] Shuai P. Liang D. Zhang Z. Yin W. Xia X. (2013). Identification of drought-responsive and novel Populus trichocarpa microRNAs by high-throughput sequencing and their targets using degradome analysis. BMC Genomics 14:233 10.1186/1471-2164-14-233 23570526 PMC3630063

[B253] Sienski G. Donertas D. Brennecke J. (2012). Transcriptional silencing of transposons by Piwi and maelstrom and its impact on chromatin state and gene expression. Cell 151, 964–980 10.1016/j.cell.2012.10.040 23159368 PMC3504300

[B254] Sijen T. Plasterk R. H. (2003). Transposon silencing in the *Caenorhabditis elegans* germ line by natural RNAi. Nature 426, 310–314 10.1038/nature02107 14628056

[B255] Skinner M. K. Haque C. G. Nilsson E. Bhandari R. Mccarrey J. R. (2013). Environmentally induced transgenerational epigenetic reprogramming of primordial germ cells and the subsequent germ line. PLoS ONE 8:e66318 10.1371/journal.pone.0066318 23869203 PMC3712023

[B256] Slaughter A. Daniel X. Flors V. Luna E. Hohn B. Mauch-Mani B. (2012). Descendants of primed Arabidopsis plants exhibit resistance to biotic stress. Plant Physiol. 158, 835–843 10.1104/pp.111.191593 22209872 PMC3271771

[B257] Slotkin R. K. (2010). The epigenetic control of the Athila family of retrotransposons in Arabidopsis. Epigenetics 5, 483–490 10.4161/epi.5.6.12119 20484989

[B258] Sokolov M. V. Dickey J. S. Bonner W. M. Sedelnikova O. A. (2007). gamma-H2AX in bystander cells: not just a radiation-triggered event, a cellular response to stress mediated by intercellular communication. Cell Cycle 6, 2210–2212 10.4161/cc.6.18.4682 17881892

[B259] Soubry A. Hoyo C. Jirtle R. L. Murphy S. K. (2014). A paternal environmental legacy: evidence for epigenetic inheritance through the male germ line. Bioessays 36, 359–371 10.1002/bies.201300113 24431278 PMC4047566

[B260] Stenberg P. Saura A. (2013). Meiosis and its deviations in polyploid animals. Cytogenet. Genome Res. 140, 185–203 10.1159/000351731 23796636

[B261] Sun G. Stewart C. N. Jr. Xiao P. Zhang B. (2012). MicroRNA expression analysis in the cellulosic biofuel crop switchgrass (*Panicum virgatum*) under abiotic stress. PLoS ONE 7:e32017 10.1371/journal.pone.0032017 22470418 PMC3314629

[B262] Sunkar R. Chinnusamy V. Zhu J. Zhu J. K. (2007). Small RNAs as big players in plant abiotic stress responses and nutrient deprivation. Trends Plant Sci. 12, 301–309 10.1016/j.tplants.2007.05.001 17573231

[B263] Suzuki S. Ono R. Narita T. Pask A. J. Shaw G. Wang C. (2007). Retrotransposon silencing by DNA methylation can drive mammalian genomic imprinting. PLoS Genet. 3:e55 10.1371/journal.pgen.0030055 17432937 PMC1851980

[B264] Svetlova M. P. Solovjeva L. V. Tomilin N. V. (2010). Mechanism of elimination of phosphorylated histone H2AX from chromatin after repair of DNA double-strand breaks. Mutat. Res. 685, 54–60 10.1016/j.mrfmmm.2009.08.001 19682466

[B265] Takahashi K. (2014). Influence of bacteria on epigenetic gene control. Cell. Mol. Life Sci. 71, 1045–1054 10.1007/s00018-013-1487-x 24132510 PMC11113846

[B266] Tang Z. Zhang L. Xu C. Yuan S. Zhang F. Zheng Y. (2012). Uncovering small RNA-mediated responses to cold stress in a wheat thermosensitive genic male-sterile line by deep sequencing. Plant Physiol. 159, 721–738 10.1104/pp.112.196048 22508932 PMC3375937

[B267] Tayale A. Parisod C. (2013). Natural pathways to polyploidy in plants and consequences for genome reorganization. Cytogenet. Genome Res. 140, 79–96 10.1159/000351318 23751271

[B268] Testori A. Caizzi L. Cutrupi S. Friard O. De Bortoli M. Cora D. (2012). The role of transposable elements in shaping the combinatorial interaction of transcription factors. BMC Genomics 13:400 10.1186/1471-2164-13-400 22897927 PMC3478180

[B269] Tian Y. Yang W. Song J. Wu Y. Ni B. (2013). Hepatitis B virus X protein-induced aberrant epigenetic modifications contributing to human hepatocellular carcinoma pathogenesis. Mol. Cell. Biol. 33, 2810–2816 10.1128/mcb.00205-13 23716588 PMC3719687

[B270] Tian Z. Yu Y. Lin F. Sanmiguel P. J. Wing R. A. Mccouch S. R. (2011). Exceptional lability of a genomic complex in rice and its close relatives revealed by interspecific and intraspecific comparison and population analysis. BMC Genomics 12:142 10.1186/1471-2164-12-142 21385395 PMC3060143

[B271] Tomas D. Bento M. Viegas W. Silva M. (2012). Involvement of disperse repetitive sequences in wheat/rye genome adjustment. Int. J. Mol. Sci. 13, 8549–8561 10.3390/ijms13078549 22942719 PMC3430250

[B272] Tsukahara S. Kawabe A. Kobayashi A. Ito T. Aizu T. Shin-I T. (2012). Centromere-targeted de novo integrations of an LTR retrotransposon of Arabidopsis lyrata. Genes Dev. 26, 705–713 10.1101/gad.183871.111 22431508 PMC3323881

[B273] Vago R. Leva V. Biamonti G. Montecucco A. (2009). DNA ligase I and Nbs1 proteins associate in a complex and colocalize at replication factories. Cell Cycle 8, 2600–2607 10.4161/cc.8.16.9352 19597347

[B274] Van Attikum H. Gasser S. M. (2005). ATP-dependent chromatin remodeling and DNA double-strand break repair. Cell Cycle 4, 1011–1014 10.4161/cc.4.8.1887 16082209

[B275] Van Rij R. P. Saleh M. C. Berry B. Foo C. Houk A. Antoniewski C. (2006). The RNA silencing endonuclease Argonaute 2 mediates specific antiviral immunity in *Drosophila melanogaster*. Genes Dev. 20, 2985–2995 10.1101/gad.1482006 17079687 PMC1620017

[B276] Vandegehuchte M. B. Janssen C. R. (2013). Epigenetics in an ecotoxicological context. Mutat. Res. [Epub ahead of print]. 10.1016/j.mrgentox.2013.08.008 24004878

[B277] Vanegas M. Llano M. Delgado S. Thompson D. Peretz M. Poeschla E. (2005). Identification of the LEDGF/p75 HIV-1 integrase-interaction domain and NLS reveals NLS-independent chromatin tethering. J. Cell Sci. 118, 1733–1743 10.1242/jcs.02299 15797927

[B278] Vanitharani R. Chellappan P. Fauquet C. M. (2005). Geminiviruses and RNA silencing. Trends Plant Sci. 10, 144–151 10.1016/j.tplants.2005.01.005 15749473

[B279] Vos L. J. Famulski J. K. Chan G. K. (2006). How to build a centromere: from centromeric and pericentromeric chromatin to kinetochore assembly. Biochem. Cell Biol. 84, 619–639 10.1139/o06-078 16936833

[B280] Wang B. Li Y. Shao C. Tan Y. Cai L. (2012). Cadmium and its epigenetic effects. Curr. Med. Chem. 19, 2611–2620 10.2174/092986712800492913 22471978

[B281] Wang G. P. Ciuffi A. Leipzig J. Berry C. C. Bushman F. D. (2007). HIV integration site selection: analysis by massively parallel pyrosequencing reveals association with epigenetic modifications. Genome Res. 17, 1186–1194 10.1101/gr.6286907 17545577 PMC1933515

[B282] Wang H. Chai Y. Chu X. Zhao Y. Wu Y. Zhao J. (2009). Molecular characterization of a rice mutator-phenotype derived from an incompatible cross-pollination reveals transgenerational mobilization of multiple transposable elements and extensive epigenetic instability. BMC Plant Biol. 9:63 10.1186/1471-2229-9-63 19476655 PMC2696445

[B283] Wang N. Wang H. Wang H. Zhang D. Wu Y. Ou X. (2010). Transpositional reactivation of the Dart transposon family in rice lines derived from introgressive hybridization with *Zizania latifolia*. BMC Plant Biol. 10:190 10.1186/1471-2229-10-190 20796287 PMC2956540

[B284] Wang T. Chen L. Zhao M. Tian Q. Zhang W. H. (2011). Identification of drought-responsive microRNAs in *Medicago truncatula* by genome-wide high-throughput sequencing. BMC Genomics 12:367 10.1186/1471-2164-12-367 21762498 PMC3160423

[B285] Wang X. Wu R. Lin X. Bai Y. Song C. Yu X. (2013a). Tissue culture-induced genetic and epigenetic alterations in rice pure-lines, F1 hybrids and polyploids. BMC Plant Biol. 13:77 10.1186/1471-2229-13-77 23642214 PMC3648424

[B286] Wang Z. H. Zhang D. Bai Y. Zhang Y. H. Liu Y. Wu Y. (2013b). Genomewide variation in an introgression line of rice-Zizania revealed by whole-genome re-sequencing. PLoS ONE 8:e74479 10.1371/journal.pone.0074479 24058573 PMC3776793

[B287] Watanabe T. Nozawa T. Aikawa C. Amano A. Maruyama F. Nakagawa I. (2013). CRISPR regulation of intraspecies diversification by limiting IS transposition and intercellular recombination. Genome Biol. Evol. 5, 1099–1114 10.1093/gbe/evt075 23661565 PMC3698921

[B288] Weber B. Heitkam T. Holtgrawe D. Weisshaar B. Minoche A. E. Dohm J. C. (2013). Highly diverse chromoviruses of Beta vulgaris are classified by chromodomains and chromosomal integration. Mob. DNA 4:8 10.1186/1759-8753-4-8 23448600 PMC3605345

[B289] Weiner A. Chen H. V. Liu C. L. Rahat A. Klien A. Soares L. (2012). Systematic dissection of roles for chromatin regulators in a yeast stress response. PLoS Biol. 10:e1001369 10.1371/journal.pbio.1001369 22912562 PMC3416867

[B290] Williams R. S. Williams J. S. Tainer J. A. (2007). Mre11-Rad50-Nbs1 is a keystone complex connecting DNA repair machinery, double-strand break signaling, and the chromatin template. Biochem. Cell Biol. 85, 509–520 10.1139/O07-069 17713585

[B291] Winkler D. D. Luger K. (2011). The histone chaperone FACT: structural insights and mechanisms for nucleosome reorganization. J. Biol. Chem. 286, 18369–18374 10.1074/jbc.R110.180778 21454601 PMC3099653

[B292] Wolfgruber T. K. Sharma A. Schneider K. L. Albert P. S. Koo D. H. Shi J. (2009). Maize centromere structure and evolution: sequence analysis of centromeres 2 and 5 reveals dynamic Loci shaped primarily by retrotransposons. PLoS Genet. 5:e1000743 10.1371/journal.pgen.1000743 19956743 PMC2776974

[B293] Wu R. Wang X. Lin Y. Ma Y. Liu G. Yu X. (2013). Inter-species grafting caused extensive and heritable alterations of DNA methylation in solanaceae plants. PLoS ONE 8:e61995 10.1371/journal.pone.0061995 23614002 PMC3628911

[B294] Xiao J. Song C. Liu S. Tao M. Hu J. Wang J. (2013). DNA methylation analysis of allotetraploid hybrids of red crucian carp (*Carassius auratus* red var.) and common carp (*Cyprinus carpio* L.). PLoS ONE 8:e56409 10.1371/journal.pone.0056409 23457564 PMC3574156

[B295] Xie W. Gai X. Zhu Y. Zappulla D. C. Sternglanz R. Voytas D. F. (2001). Targeting of the yeast Ty5 retrotransposon to silent chromatin is mediated by interactions between integrase and Sir4p. Mol. Cell. Biol. 21, 6606–6614 10.1128/MCB.21.19.6606-6614.2001 11533248 PMC99806

[B296] Xin M. Wang Y. Yao Y. Song N. Hu Z. Qin D. (2011). Identification and characterization of wheat long non-protein coding RNAs responsive to powdery mildew infection and heat stress by using microarray analysis and SBS sequencing. BMC Plant Biol. 11:61 10.1186/1471-2229-11-61 21473757 PMC3079642

[B297] Xin M. Wang Y. Yao Y. Xie C. Peng H. Ni Z. (2010). Diverse set of microRNAs are responsive to powdery mildew infection and heat stress in wheat (*Triticum aestivum* L.). BMC Plant Biol. 10:123 10.1186/1471-2229-10-123 20573268 PMC3095282

[B298] Xiong Z. Gaeta R. T. Pires J. C. (2011). Homoeologous shuffling and chromosome compensation maintain genome balance in resynthesized allopolyploid *Brassica napus*. Proc. Natl. Acad. Sci. U.S.A. 108, 7908–7913 10.1073/pnas.1014138108 21512129 PMC3093481

[B299] Xu L. Wang Y. Zhai L. Xu Y. Wang L. Zhu X. (2013). Genome-wide identification and characterization of cadmium-responsive microRNAs and their target genes in radish (*Raphanus sativus* L.) roots. J. Exp. Bot. 64, 4271–4287 10.1093/jxb/ert240 24014874 PMC3808317

[B300] Xu Y. Ayrapetov M. K. Xu C. Gursoy-Yuzugullu O. Hu Y. Price B. D. (2012a). Histone H2A.Z controls a critical chromatin remodeling step required for DNA double-strand break repair. Mol. Cell 48, 723–733 10.1016/j.molcel.2012.09.026 23122415 PMC3525728

[B301] Xu Y. Huang L. Fu S. Wu J. Zhou X. (2012b). Population diversity of rice stripe virus-derived siRNAs in three different hosts and RNAi-based antiviral immunity in Laodelphgax striatellus. PLoS ONE 7:e46238 10.1371/journal.pone.0046238 23029445 PMC3460854

[B302] Xu Y. Price B. D. (2011). Chromatin dynamics and the repair of DNA double strand breaks. Cell Cycle 10, 261–267 10.4161/cc.10.2.14543 21212734 PMC3048797

[B303] Xu Z. Zhong S. Li X. Li W. Rothstein S. J. Zhang S. (2011). Genome-wide identification of microRNAs in response to low nitrate availability in maize leaves and roots. PLoS ONE 6:e28009 10.1371/journal.pone.0028009 22132192 PMC3223196

[B304] Yaakov B. Kashkush K. (2011a). Massive alterations of the methylation patterns around DNA transposons in the first four generations of a newly formed wheat allohexaploid. Genome 54, 42–49 10.1139/g10-091 21217805

[B305] Yaakov B. Kashkush K. (2011b). Methylation, transcription, and rearrangements of transposable elements in synthetic allopolyploids. Int. J. Plant Genomics 2011:569826 10.1155/2011/569826 21760771 PMC3134107

[B306] Yaakov B. Kashkush K. (2012). Mobilization of Stowaway-like MITEs in newly formed allohexaploid wheat species. Plant Mol. Biol. 80, 419–427 10.1007/s11103-012-9957-3 22933118

[B307] Yang L. Jue D. Li W. Zhang R. Chen M. Yang Q. (2013). Identification of MiRNA from eggplant (*Solanum melongena* L.) by small RNA deep sequencing and their response to *Verticillium dahliae* infection. PLoS ONE 8:e72840 10.1371/journal.pone.0072840 24015279 PMC3754920

[B308] Yang N. Kazazian H. H. Jr. (2006). L1 retrotransposition is suppressed by endogenously encoded small interfering RNAs in human cultured cells. Nat. Struct. Mol. Biol. 13, 763–771 10.1038/nsmb1141 16936727

[B309] Yang X. Xie Y. Raja P. Li S. Wolf J. N. Shen Q. (2011). Suppression of methylation-mediated transcriptional gene silencing by betaC1-SAHH protein interaction during geminivirus-betasatellite infection. PLoS Pathog. 7:e1002329 10.1371/journal.ppat.1002329 22028660 PMC3197609

[B310] Yang X. Yu Y. Jiang L. Lin X. Zhang C. Ou X. (2012). Changes in DNA methylation and transgenerational mobilization of a transposable element (mPing) by the topoisomerase II inhibitor, etoposide, in rice. BMC Plant Biol. 12:48 10.1186/1471-2229-12-48 22482475 PMC3480845

[B311] Yao Y. Danna C. H. Zemp F. J. Titov V. Ciftci O. N. Przybylski R. (2011). UV-C-irradiated Arabidopsis and tobacco emit volatiles that trigger genomic instability in neighboring plants. Plant Cell 23, 3842–3852 10.1105/tpc.111.089003 22028460 PMC3229153

[B312] Yao Y. Kathiria P. Kovalchuk I. (2013). A systemic increase in the recombination frequency upon local infection of *Arabidopsis thaliana* plants with oilseed rape mosaic virus depends on plant age, the initial inoculum concentration and the time for virus replication. Front. Plant Sci. 4:61 10.3389/fpls.2013.00061 23519399 PMC3604634

[B313] Yao Y. Kovalchuk I. (2011). Abiotic stress leads to somatic and heritable changes in homologous recombination frequency, point mutation frequency and microsatellite stability in Arabidopsis plants. Mutat. Res. 707, 61–66 10.1016/j.mrfmmm.2010.12.013 21236268

[B314] Yap K. L. Zhou M. M. (2011). Structure and mechanisms of lysine methylation recognition by the chromodomain in gene transcription. Biochemistry 50, 1966–1980 10.1021/bi101885m 21288002 PMC3062707

[B315] Ye Y. H. Woolfit M. Huttley G. A. Rances E. Caragata E. P. Popovici J. (2013). Infection with a virulent strain of disrupts genome wide-patterns of cytosine methylation in the mosquito. PLoS ONE 8:e66482 10.1371/journal.pone.0066482 23840485 PMC3686743

[B316] Yin Z. Li Y. Han X. Shen F. (2012). Genome-wide profiling of miRNAs and other small non-coding RNAs in the *Verticillium dahliae*-inoculated cotton roots. PLoS ONE 7:e35765 10.1371/journal.pone.0035765 22558219 PMC3338460

[B317] Youngson N. A. Kocialkowski S. Peel N. Ferguson-Smith A. C. (2005). A small family of sushi-class retrotransposon-derived genes in mammals and their relation to genomic imprinting. J. Mol. Evol. 61, 481–490 10.1007/s00239-004-0332-0 16155747

[B318] Yu S. Teng Y. Waters R. Reed S. H. (2011). How chromatin is remodelled during DNA repair of UV-induced DNA damage in *Saccharomyces cerevisiae*. PLoS Genet. 7:e1002124 10.1371/journal.pgen.1002124 21698136 PMC3116912

[B319] Yu Y. Deng Y. Reed S. H. Millar C. B. Waters R. (2013a). Histone variant Htz1 promotes histone H3 acetylation to enhance nucleotide excision repair in Htz1 nucleosomes. Nucleic Acids Res. 41, 9006–9019 10.1093/nar/gkt688 23925126 PMC3799447

[B320] Yu Y. Yang X. Wang H. Shi F. Liu Y. Liu J. (2013b). Cytosine methylation alteration in natural populations of *Leymus chinensis* induced by multiple abiotic stresses. PLoS ONE 8:e55772 10.1371/journal.pone.0055772 23418457 PMC3572093

[B321] Zeng Q. Y. Yang C. Y. Ma Q. B. Li X. P. Dong W. W. Nian H. (2012). Identification of wild soybean miRNAs and their target genes responsive to aluminum stress. BMC Plant Biol. 12:182 10.1186/1471-2229-12-182 23040172 PMC3519564

[B322] Zhai L. Liu Z. Zou X. Jiang Y. Qiu F. Zheng Y. (2013). Genome-wide identification and analysis of microRNA responding to long-term waterlogging in crown roots of maize seedlings. Physiol. Plant. 147, 181–193 10.1111/j.1399-3054.2012.01653.x 22607471

[B323] Zhan X. Wang B. Li H. Liu R. Kalia R. K. Zhu J. K. (2012). Arabidopsis proline-rich protein important for development and abiotic stress tolerance is involved in microRNA biogenesis. Proc. Natl. Acad. Sci. U.S.A. 109, 18198–18203 10.1073/pnas.1216199109 23071326 PMC3497810

[B324] Zhang W. Gao S. Zhou X. Chellappan P. Chen Z. Zhou X. (2011). Bacteria-responsive microRNAs regulate plant innate immunity by modulating plant hormone networks. Plant Mol. Biol. 75, 93–105 10.1007/s11103-010-9710-8 21153682 PMC3005105

[B325] Zhang W. Lee H. R. Koo D. H. Jiang J. (2008a). Epigenetic modification of centromeric chromatin: hypomethylation of DNA sequences in the CENH3-associated chromatin in *Arabidopsis thaliana* and maize. Plant Cell 20, 25–34 10.1105/tpc.107.057083 18239133 PMC2254920

[B326] Zhang X. Ge X. Shao Y. Sun G. Li Z. (2013). Genomic change, retrotransposon mobilization and extensive cytosine methylation alteration in *Brassica napus* introgressions from two intertribal hybridizations. PLoS ONE 8:e56346 10.1371/journal.pone.0056346 23468861 PMC3585313

[B327] Zhang Y. Mager D. L. (2012). Gene properties and chromatin state influence the accumulation of transposable elements in genes. PLoS ONE 7:e30158 10.1371/journal.pone.0030158 22272293 PMC3260225

[B328] Zhang Z. Lin H. Shen Y. Gao J. Xiang K. Liu L. (2012). Cloning and characterization of miRNAs from maize seedling roots under low phosphorus stress. Mol. Biol. Rep. 39, 8137–8146 10.1007/s11033-012-1661-5 22562381 PMC3383953

[B329] Zhang Z. Wei L. Zou X. Tao Y. Liu Z. Zheng Y. (2008b). Submergence-responsive MicroRNAs are potentially involved in the regulation of morphological and metabolic adaptations in maize root cells. Ann. Bot. 102, 509–519 10.1093/aob/mcn129 18669574 PMC2701776

[B330] Zheng X. Chen L. Li M. Lou Q. Xia H. Wang P. (2013). Transgenerational variations in DNA methylation induced by drought stress in two rice varieties with distinguished difference to drought resistance. PLoS ONE 8:e80253 10.1371/journal.pone.0080253 24244664 PMC3823650

[B331] Zheng Y. Ao Z. Jayappa K. D. Yao X. (2010). Characterization of the HIV-1 integrase chromatin- and LEDGF/p75-binding abilities by mutagenic analysis within the catalytic core domain of integrase. Virol. J. 7:68 10.1186/1743-422x-7-68 20331877 PMC2859858

[B332] Zhou M. Li D. Li Z. Hu Q. Yang C. Zhu L. (2013). Constitutive expression of a miR319 gene alters plant development and enhances salt and drought tolerance in transgenic creeping bentgrass. Plant Physiol. 161, 1375–1391 10.1104/pp.112.208702 23292790 PMC3585603

[B333] Zhu H. Guo H. (2012). The role of virus-derived small interfering RNAs in RNA silencing in plants. Sci. China Life Sci. 55, 119–125 10.1007/s11427-012-4281-3 22415682

[B334] Zhu J. Gaiha G. D. John S. P. Pertel T. Chin C. R. Gao G. (2012). Reactivation of latent HIV-1 by inhibition of BRD4. Cell Rep. 2, 807–816 10.1016/j.celrep.2012.09.008 23041316 PMC3523124

[B335] Zhu Y. Rowley M. J. Bohmdorfer G. Wierzbicki A. T. (2013). A SWI/SNF chromatin-remodeling complex acts in noncoding RNA-mediated transcriptional silencing. Mol. Cell 49, 298–309 10.1016/j.molcel.2012.11.011 23246435 PMC3560041

[B336] Zhuang Y. Zhou X. H. Liu J. (2014). Conserved miRNAs and their response to salt stress in wild Eggplant *Solanum linnaeanum* roots. Int. J. Mol. Sci. 15, 839–849 10.3390/ijms15010839 24413753 PMC3907842

[B337] Zucchi F. C. Yao Y. Metz G. A. (2012). The secret language of destiny: stress imprinting and transgenerational origins of disease. Front. Genet. 3:96 10.3389/fgene.2012.00096 22675331 PMC3366387

[B338] Zvereva A. S. Pooggin M. M. (2012). Silencing and innate immunity in plant defense against viral and non-viral pathogens. Viruses 4, 2578–2597 597. 10.3390/v4112578 23202495 PMC3509663

